# Renal Outcomes Over the Course of 5 Years of Oral HIV Preexposure Prophylaxis Using Tenofovir Disoproxil/Emtricitabine

**DOI:** 10.1016/j.ekir.2025.03.033

**Published:** 2025-03-25

**Authors:** Dita C. Bolluyt, Mark A.M. van den Elshout, Eline S. Wijstma, Anders Boyd, Elske Hoornenborg, Henry J.C. De Vries, Maria Prins, Liffert Vogt, Maarten F. Schim van der Loeff, Amsterdam PrEP Project Team in the HIV Transmission Elimination AMsterdam Initiative (H-TEAM), T. van Benthem, D. Bons, G.J. de Bree, P. Brokx, U. Davidovich, S. Hendriks, S.E. Geerlings, M. Heidenrijk, E. Hoornenborg, J. Heijne, P. Reiss, A. van Sighem, M. van der Valk, J. de Wit, P. Zantkuijl, N. Schat, L. Dol, M. van Agtmael, J. Ananworanich, D. Van de Beek, G.E.L. van den Berk, D. Bezemer, A. van Bijnen, J.P. Bil, W.L. Blok, S. Bogers, M. Bomers, A. Boyd, W. Brokking, D. Burger, K. Brinkman, N. Brinkman, M. de Bruin, S. Bruisten, L. Coyer, R. van Crevel, M. Dijkstra, Y.T. van Duijnhoven, A. van Eeden, L. Elsenburg, M.A.M. van den Elshout, E. Ersan, P.E.V. Felipa, T.B.H. Geijtenbeek, J. van Gool, A. Goorhuis, M. Groot, C.A. Hankins, A. Heijnen, M.M.J. Hillebregt, M. Hommenga, J.W. Hovius, Y. Janssen, K. de Jong, V. Jongen, N.A. Kootstra, R.A. Koup, F.P. Kroon, T.J.W. van de Laar, F. Lauw, M.M. van Leeuwen, K. Lettinga, I. Linde, D.S.E. Loomans, I.M. van der Lubben, J.T. van der Meer, T. Mouhebati, B.J. Mulder, J. Mulder, F.J. Nellen, A. Nijsters, H. Nobel, E.L.M. Op de Coul, E. Peters, I.S. Peters, T. van der Poll, O. Ratmann, C. Rokx, M.F. Schim van der Loeff, W.E.M. Schoute, J. Schouten, J. Veenstra, A. Verbon, F. Verdult, J. de Vocht, H.J. de Vries, S. Vrouenraets, M. van Vugt, W.J. Wiersinga, F.W. Wit, L.R. Woittiez, S. Zaheri, P. Zantkuijl, Żakowicz, M.C. van Zelm, H.M.L. Zimmermann

**Affiliations:** 1 Department of Infectious Diseases, Public Health Service of Amsterdam, Amsterdam, The Netherlands; 2 Stichting HIV Monitoring, Amsterdam, The Netherlands; 3 Amsterdam Institute for Immunology and Infectious Diseases, Amsterdam UMC, University of Amsterdam, Amsterdam, The Netherlands; 4 Department of Dermatology, Amsterdam UMC location University of Amsterdam, Amsterdam, The Netherlands; 5 Department of Infectious Diseases, Amsterdam UMC, University of Amsterdam, Amsterdam, The Netherlands; 6 Department of Nephrology, Amsterdam UMC, University of Amsterdam, Amsterdam, The Netherlands

**Keywords:** eGFR, HIV, kidney function, men who have sex with men, preexposure prophylaxis

## Abstract

**Introduction:**

Tenofovir disoproxil fumarate/emtricitabine (TDF/FTC) is used as oral preexposure prophylaxis (PrEP) to prevent HIV. Because TDF could be nephrotoxic, we assessed the association between PrEP use and kidney function over time.

**Methods:**

We included men who have sex with men (MSM) from the Amsterdam PrEP demonstration Project (AMPrEP; 2015–2020) at the Public Health Service of Amsterdam, who had an estimated glomerular filtration rate (eGFR) ≥ 60 ml/min per 1.73 m2 and ≥ 1 creatinine measurement after baseline. Participants could choose between daily or event-driven PrEP use. Plasma creatinine was measured at PrEP commencement and annually thereafter. Kidney function was calculated as the eGFR using the 2021 Chronic Kidney Disease-Epidemiology Collaboration formula.

**Results:**

Among the 351 participants analyzed (median age: 41 years, interquartile range [IQR] = 33–49), mean eGFR at PrEP commencement was 100 ml/min per 1.73 m2 (SD = 14). During a median follow-up of 54.2 months (IQR = 47.0–57.6), eGFR decreased by 0.30/yr (95% confidence interval [CI] = − 0.59 to − 0.01). We observed lower mean eGFR decline in those using daily PrEP than in those using event-driven PrEP (− 3.05, 95% CI = − 3.95 to − 2.15), and in older participants (− 5.75/10 yrs, 95% CI = − 6.70 to − 4.80). Daily PrEP users had an average decline in eGFR of 0.57 ml/min per 1.73 m2/yr (95% CI = − 1.06 to − 0.08), and there was no statistically significant decline in event-driven PrEP users (P for interaction = 0.30). Twelve participants (3.4%) had an incident eGFR < 60 during follow-up, none of which persisted.

**Conclusion:**

Daily PrEP users had a faster decline over time than event-driven PrEP users, yet eGFR declines were low and within the range of those expected in the general population. Our data support reduced kidney function monitoring in PrEP users at low risk for kidney dysfunction.

## Graphical abstract

**Figure:**
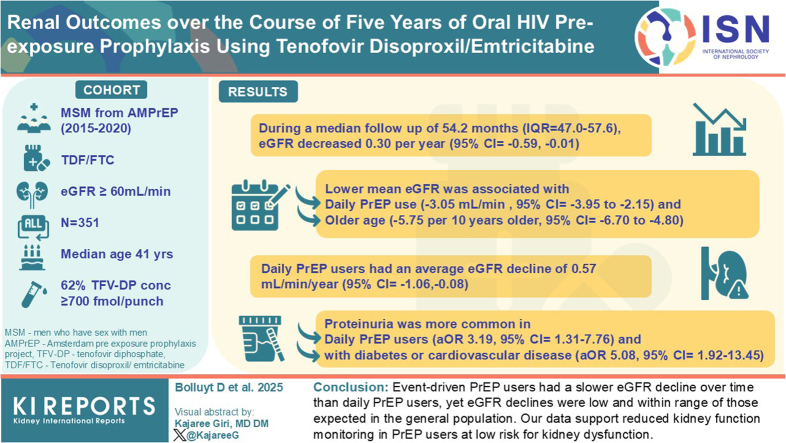

TDF/FTC has been proven effective since 2010 as oral PrEP for prevention of sexual HIV transmission.^1^ The global prevalence of HIV is 7.7% higher among MSM than among other adults^2^; thus, MSM are considered a key population at higher risk for HIV. HIV prevention is then important for MSM who engage in activities associated with HIV acquisition and could be assisted by optimal uptake and persistent use of PrEP.

There are several crucial barriers that prevent not only people from using PrEP, but also practitioners from prescribing PrEP. One such example is the high frequency of regular medical follow-ups,^3^ which includes screening for kidney damage.^4^ Several case reports and cohort studies have shown an association between cumulative TDF exposure in HIV treatment and nephrotoxicity, in particular of the proximal tubule.^5^ Although TDF/FTC use for PrEP is considered safe and well-tolerated, some studies have shown a statistically significant decline in eGFR, which was reversible after discontinuation of PrEP.^6^^,^^7^ A recently published meta-analysis found that among 14,368 PrEP users, 2.4% had a decline in eGFR to < 60 ml/min per 1.73 m^2^ after PrEP-commencement.^8^ The risk of a clinically significant decline in kidney function is known to increase with age. PrEP guidance from the World Health Organization therefore suggests that kidney function measurement can be considered optional in individuals aged ≤ 30 years and should be performed at least yearly among people with comorbidities and those aged ≥ 50 years.^4^ Despite this recommendation, many of the previous studies have limited follow-up (i.e., median of only 10 months), and no data were available on daily versus event-driven PrEP use. Long-term data are needed to comprehensively address the role of cumulative PrEP exposure in the development of adverse kidney events. The aim of this study was to assess the association between kidney function and oral PrEP use over 5 years among daily and event-driven PrEP users in the Amsterdam PrEP demonstration project (AMPrEP) prospective cohort.

## Methods

### Study Setting

AMPrEP was a prospective, longitudinal, open-label study with recruitment between August 2015 and May 2016, and follow-up until December 2020. The main aims were to assess the uptake of PrEP among PrEP-eligible MSM and transgender women and to assess PrEP adherence and the incidence of sexually transmitted infections. The study design has been published elsewhere.^9^ In brief, participants were eligible for inclusion if they were HIV-negative MSM or transgender persons, aged ≥18 years, and reported at least 1 risk factor for HIV acquisition in the 6 months before the PrEP screening visit. Exclusion criteria were eGFR < 60 ml/min per 1.73 m^2^ (calculated with Cockcroft-Gault formula), or concurrent use of nephrotoxic medication. Participants attended follow-up visits every 3 months. Restarting participation was allowed. We offered participants a choice between daily PrEP or event-driven PrEP; they could switch regimens at each study visit.

For the present study, participants were excluded from the analyses if they discontinued or were lost to follow-up before creatinine measurement at the 12-month visit and did not restart. None of the AMPrEP participants discontinued before the first creatinine measurement because of kidney injury.

### Exposures and Outcomes

We used baseline demographic data (i.e., age, gender identity, self-declared ethnicity, and socioeconomic status). Sex was defined as sex assigned at birth; gender was defined as current gender identity. PrEP regimen, categorized as daily or event-driven PrEP, was included as a time-varying variable. We also used data of factors that could potentially influence kidney function, all recorded annually, including the following: the Alcohol Use Disorders Identification Test (AUDIT, score ≥ 8 indicates alcohol use disorder^10^), recreational drug use (e.g., amphetamine, cannabis, cocaine, GHB, and XTC/MDMA) during sex, and the Drug Use Disorders Identification Test (DUDIT, score ≥ 8 indicates drug use disorder^11^). Comedication (including over-the-counter), were collected at every study visit. We tested for hepatitis C virus infection annually until December 2016, and every 6 months thereafter. We measured plasma creatinine at baseline and annually, and performed dipstick urinalysis (UroColor 3, SD, Korea) to measure proteinuria at every study visit. Persistent proteinuria was defined as ≥ 2 consecutive measurements with ≥ 1+.

We categorized concomitant medication use as nephrotoxic, nonnephrotoxic, or no comedication. Medication was considered to be nephrotoxic if the Liverpool HIV drug interactions database indicated a potential effect on kidney function.^12^ Medication was considered to be nonnephrotoxic if the database indicated a weak effect. If the medication was not registered, the Dutch medication database^13^ was consulted. Participants who were treated with antihypertensives, diuretics, beta-blockers, antiarrythmics, anticoagulants or lipid-modifying agents were considered to have cardiovascular disease. Participants who reported the use of insulin or oral blood glucose–lowering agents were considered to be diabetic.

Dried bloodspots were collected to measure intraerythrocytic tenofovir diphosphate (TFV-DP) concentrations at 12-, 24-, and 48-month visits. A detailed description of the laboratory methods has been previously published.^14^ Good adherence was defined as a TFV-DP concentration ≥ 700 fmol/punch (corresponding to ≥ 4 tablets/wk on average).^15^

Plasma creatinine concentration (in μmol/l) was determined using an enzymatic method at the Cobas 8000 c702 platform (Roche, Basel, Switzerland). We calculated eGFR using the 2021 Chronic Kidney Disease-Epidemiology Collaboration formula.^16^ Kidney function was defined as normal (eGFR ≥ 90 ml/min per 1.73 m^2^) and impaired when < 60 ml/min per 1.73 m^2^ following the guidelines from the Kidney Disease: Improving Global Outcomes Group.^17^ We measured proteinuria as negative, trace (∼10 mg/dl), 1+ (∼30 mg/dl), 2+ (∼100 mg/dl), 3+ (∼300 mg/dl), or 4+ (∼1000 mg/dl).

### Statistical Analysis

Baseline was defined as the enrolment study visit. Follow-up began at baseline and continued until study discontinuation, loss to follow-up, HIV diagnosis, or administrative censoring, whichever occurred first. We compared the characteristics of daily versus event-driven PrEP users at baseline using *t* test or Mann Whitney U-test for continuous variables and Pearson’s χ^2^ or Fisher Exact test for categorical variables. The following yearly recorded variables were carried forward to study visits during which they were not measured: AUDIT, DUDIT, and recreational drug use.

We modeled the mean eGFR using generalized estimating equations for linear regression with an exchangeable working correlation to correct for repeated measurements. Generalized estimating equations were necessary because participants could switch from event-driven PrEP to daily PrEP or vice versa, so were not necessarily in the same exposure group at each measurement. In a determinant analysis, we included covariates to the model to estimate the mean difference across levels of determinants and their 95% CIs. Candidate determinants included PrEP use and risk factors for chronic kidney disease. Time (in study), age at baseline, and having diabetes or cardiovascular disease were selected *a priori*. Other determinants were selected in a stepwise manner. First, we included variables with *P* < 0.25 in univariable analyses via forward selection. We then removed variables with a *P* ≥ 0.05 via backward selection. Finally, excluded variables in the initial, forward-stepwise selection were reconsidered in the preliminary model again using forward selection if *P* < 0.05. Because of collinearity between PrEP regimen and TFV-DP concentration, we created 2 separate multivariable models, one including PrEP regimen and the other including TFV-DP concentration as a covariate.

We modeled the probability of having proteinuria using generalized estimating equations for logistic regression with exchangeable working correlation. We included covariates separately to estimate the odds ratio and its 95% CI comparing the odds of proteinuria across levels of variables.

We constructed a multivariable model in which we included *a priori* having diabetes or cardiovascular disease. eGFR was carried forward to study visits during which it was not measured. We included variables with a *P* < 0.25 in univariable analysis via forward stepwise selection and removed variables with a *P* ≥ 0.05 in backward-stepwise-selection.

We performed several sensitivity analyses. To assess the possible effect of selective loss to follow-up related to kidney function, we reran the analysis of determinants of eGFR among participants with at least 5 years of follow-up. Because participants switching PrEP regimens could have different changes in eGFR, depending on cumulative exposure to TDF/FTC during follow-up and the ordering of time on daily and event-driven PrEP, we also reran the analysis among participants who never switched regimens. To enable comparisons with previous studies,^8^ we performed the analysis on the determinants of changes in mean eGFR calculated with the Cockcroft-Gault formula.

Characteristics of participants whose eGFR became < 60 ml/min per 1.73 m^2^ during study follow-up and of participants who had persistent proteinuria (defined as ≥ 2 consecutive visits at which proteinuria was found) were described.

We defined significance as *P* < 0.05. Multivariable analyses were based on complete case analysis. All analyses were performed using STATA Intercooled 17 (STATA Corporation, College Station, TX).

### Ethical Approval

The study was approved by the ethics board of the Academic Medical Center, Amsterdam, The Netherlands (NL49504.018.14) and is registered at the Dutch trial registry (NTR5411).

## Results

### Description of Study Population at Baseline

Of 376 participants initiating PrEP, 351 had a follow-up plasma creatinine measurement and were included in the analyses (Table S1). Two participants identified as transgender women and 349 as male (Table 1). Median age was 41 years (IQR =33–49), event-driven PrEP users were older than daily PrEP users (median of 45 vs. 38 years). Most participants self-declared as White (84.9%) and had a university or university of applied sciences degree (*n* = 271, 77%). A small proportion of participants had diabetes or cardiovascular disease (*n* = 8 [2%] and *n* = 48 [14%], respectively). People with diabetes or cardiovascular disease had a significantly higher weight than those without the same (*P* = 0.001). Most participants did not use any comedication at baseline (*n* = 207, 59%), whereas 20 (6%) used potentially nephrotoxic comedications. At baseline, 96 participants (27%) chose event-driven PrEP.

**Table 1:** Baseline characteristics measured at study enrolment (2015–2016) and follow-up time (2015–2020) of AMPrEP participants by PrEP regimen, Amsterdam, The Netherlands

Characteristics	Total (*N* = 351)		Daily PrEP (*n* = 256)		Event-driven PrEP (*n* = 95)		*P*-value
	*n*	%^a^	*n*	%^a^	*n*	%^a^	
Demographic characteristics							
Age (yrs)							< 0.001
Median (IQR)	41	(33–49)	38	(32–48)	45	(35–53)	
Age (yrs, categorized)							0.005
< 30	63	18%	54	21%	9	9%	
30–39	105	30%	82	32%	23	24%	
40–49	96	27%	66	26%	30	32%	
≥ 50	87	25%	54	21%	33	35%	
Gender identity							0.47
Male	349	99%	255	99%	94	99%	
Transgender woman	2	1%	1	1%	1	1%	
Self-declared ethnicity							0.90
White	300	85%	214	84%	85	89%	
Arabic	1	0%	1	0%	0	0%	
Asian	10	3%	8	3%	2	2%	
Black African	6	2%	5	2%	1	1%	
Black Other	5	1%	5	2%	0	0%	
Mixed	24	7%	18	7%	6	6%	
Other	5	1%	4	2%	1	1%	
Education level							0.06
No university/university for applied sciences	80	23%	65	25%	15	16%	
University/university for applied sciences	271	77%	191	75%	80	84%	
Employment^b^							0.18
Employed	270	78%	198	79%	72	76%	
Unemployed	17	5%	9	4%	8	8%	
Other (retired, volunteer, disabled, student)	60	17%	45	18%	15	16%	
Net monthly income in Euro^c^							0.53
≤ 1700	92	27%	68	28%	24	26%	
1701–2950	144	43%	106	44%	38	40%	
> 2950	99	30%	67	28%	32	34%	
Variables that may influence renal function							
Creatinine (μmol/l)							0.08
Mean (SD)	86	(12)	87	(13)	84	(12)	
eGFR, CKD-EPI 2021, ml/min per 1.73 m^2^							0.45
Mean (SD)	100	(14)	100	(15)	99	(13)	
KDIGO kidney function stage							0.92
Normal ≥ 90 ml/min per 1.73 m^2^	261	74%	191	75%	70	74%	
Mild impairment, 60–89 ml/min per 1.73 m^2^	89	25%	64	25%	25	26%	
Moderate impairment, 30–59 ml/min per 1.73 m^2^	1	0,3%	1	0,3%	0	0%	
Proteinuria, dipstick^c^^,^^d^							0.83
Negative	283	84%	207	83%	76	86%	
Trace	50	15%	39	16%	11	13%	
≥+1	4	1%	3	1%	1	1%	
AUDIT^e^							0.36
Score < 8 (no indication)^f^	253	73%	181	72%	72	76%	
Score ≥ 8 (indication)^g^	95	27%	72	28%	23	24%	
DUDIT							0.16
Score < 8 (no indication)^h^	223	64%	157	61%	66	69%	
Score ≥ 8 (indication)^i^	128	36%	99	38%	29	31%	
Drug use during sex in the past 6 mo^j^							0.99
No	159	45%	116	46%	43	46%	
Yes	184	54%	134	54%	50	54%	
Current medicine use							0.22
No	207	59%	146	57%	61	64%	
Yes, not nephrotoxic	124	35%	97	38%	27	28%	
Yes, (potentially) nephrotoxic	20	6%	13	5%	7	7%	
Diabetes mellitus^k^	8	2%	4	2%	4	4%	0.22
Cardiovascular disease^k^	48	14%	32	13%	16	17%	0.30
Weight (kg)							
Total mean (SD)	82	(14)	81	(14)	83	(15)	0.40
among participants with CVD or diabetes	89	(15)					0.0001^l^
among other participants	81	(13)					
HCV status^m^							0.77
HCV-RNA positive	335	96%	243	96%	92	97%	
HCV-RNA negative	14	4%	11	4%	3	3%	
Follow-up							
Time of follow-up, mos							0.55
Median (IQR)	54	(47–58)	54	(47–58)	54	(47–57)	

AMPrEP, Amsterdam PrEP demonstration project; AUDIT, alcohol use disorder identification test; CKD-EPI, Chronic Kidney Disease - Epidemiology Collaboration; CVD, cardiovascular disease; DUDIT, drug use disorder identification test; eGFR, estimated glomerular filtration rate (using the CKD-EPI 2021 equation); HCV, hepatitis C virus; IQR, interquartile range; KDIGO, Kidney Disease: Improving Global Outcomes Group; PrEP, preexposure prophylaxis.

*P*-values for continuous variables calculated with *t* test or Mann Whitney U-test, for categorical variables with Pearson’s Chi-square test or a Fishers exact test (*n* < 5).

a Percentages may not total 100 because of rounding.

b 4 missing.

c 16 missing.

d Measured at first study visit (1 month after enrolment).

e 3 missing.

f No indication of an alcohol use disorder.

g Indication of an alcohol use disorder.

h No indication of a drug use disorder.

i Indication of a drug use disorder.

j 8 missing.

k Deduced from medication use.

l Comparing weight between participants with and without CVD or diabetes.

m 2 missing.

At baseline and before PrEP initiation, mean eGFR was 100 ml/min per 1.73 m^2^ (SD = 14). Ninety participants (25%) had an impaired kidney function, among whom 1 participant with an eGFR < 60 ml/min per 1.73 m^2^. This participant was not excluded, because the eGFR as per the Cockcroft-Gault formula used at inclusion was 100 ml/min per 1.73 m^2^, and eGFR during follow-up remained > 60 ml/min per 1.73 m^2^. There was no difference in kidney function between event-driven and daily PrEP users. Participants with an impaired kidney function were older than those with a normal kidney function (Table S2). Four participants (1.2%) had proteinuria at the first measurement (i.e., 1 month after PrEP initiation).

### Description of Follow-Up

Median follow-up time was 54.2 months (IQR = 47.0–57.6) and 261 (74.4%) participants had a follow-up duration of at least 48 months. Mean eGFR at 48 months was 96 ml/min per 1.73 m^2^ (SD = 15). Two hundred one participants (57.3%) never switched PrEP regimens. During follow-up 2096 serum creatinine measurements were performed, with a median of 6 (IQR = 6–7) measurements per participant. A total of 5642 urine dipstick measurements were performed; 5041 (89%) were negative for proteinuria, 520 (9%) showed a trace of protein, and 81 (1%) were positive (Figure S3). The median number of urine dipstick measurements per participant was 18 (IQR = 16–19). Among daily PrEP users, median TFV-DP concentration at 3 or 6 months was 1173 fmol/punch (IQR = 864–1548; *n* = 285); at 12 months, it was 1351 fmol/punch (IQR = 1103–1697; *n* = 251); at 24 months, it was 1288 fmol/punch (IQR = 1005–1617; *n* = 223); and at 48 months, it was 1693 fmol/punch (IQR = 1310–2252; *n* = 127). Seven hundred fifty-two of 1216 of TFV-DP measurements (62%) were ≥ 700 fmol/punch.

### Renal Impairment During PrEP Use

In univariable analysis, lower mean eGFR was observed with higher age at baseline, longer follow-up, using daily PrEP, and having diabetes or cardiovascular disease. Conversely, higher mean eGFR was found in those with non-White ethnicity and a DUDIT score ≥ 8 (Table 2). In multivariable analysis including PrEP regimen, lower mean eGFR was associated with daily PrEP (− 3.02 ml/min per 1.73 m^2^, 95% CI = − 3.92 to − 2.19) and higher age at baseline (− 6.16 ml/min per 1.73 m^2^/10 yrs older, 95% CI = − 7.17 to − 5.14; Table 2). In the multivariable model including TFV-DP concentration rather than PrEP regimen, eGFR was associated with TFV-DP concentration (− 0.18 ml/min per 1.73 m^2^ per 100 fmol/punch increase, 95% CI = − 0.31 to − 0.05) and higher age at baseline (− 5.77 ml/min per 1.73 m^2^/10 yrs older, 95% CI = − 6.84 to − 4.70).

**Table 2:** Determinants of eGFR (using the CKD-EPI 2021 equation) in linear regression using GEE among AMPrEP participants (*N* = 351, number of creatinine measures = 2096), Amsterdam, The Netherlands 2015–2020

Characteristics	Number of measurements (%)	Univariable model		Multivariable model (including PrEP regimen [*n* = 2094])		Multivariable model (including TFV-DP concentration [*n* = 832]^a^)	
		difference in eGFR in ml/min per 1.73 m^2^ (95% CI)	*P*-value	difference in eGFR in ml/min per 1.73 m^2^ (95% CI)	*P*-value	difference in eGFR in mL/min/1.73 m^2^ (95% CI)	*P*-value
Demographic characteristics							
Age at baseline (per 10 yrs older)		−5.75 (−6.70 to −4.80)	<0.0001	−6.16 (−7.17 to −5.14)	<0.0001	−5.77 (−6.84 to −4.70)	<0.0001
Self-declared ethnicity, not White	290 (14%)	5.63 (1.66 to 9.60)	0.006				
PrEP use							
Time of follow-up, per additional yr		−0.30 (−0.59 to −0.01)	0.04	−0.04 (−0.36 to 0.27)	0.79	−0.58 (−1.24 to 0.90)	0.09
PrEP regimen^b^							
Daily	1273 (61%)	−3.05 (−3.95 to −2.15)	<0.0001	−3.02 (−3.92 to −2.19)	<0.0001		
Event-driven	472 (23%)	−0.59 (−1.93 to 0.75)	0.39	−0.36 (−1.79 to 1.06)	0.62		
TFV-DP concentration (per 100 fmol/punch higher)^a^	832	−0.11 (−0.20 to −0.03)	0.01			−0.18 (−0.31 to −0.05)	0.006
Variables that may influence kidney function							
AUDIT^c^							
Score ≥ 8 (indication)^d^	478 (23%)	0.89 (−0.65 to 2.44)	0.26				
DUDIT^e^							
Score ≥ 8 (indication)^f^	625 (30%)	1.34 (0.12–2.55)	0.03				
Drug use during sex in the past 6 mo^g^	965 (46%)	−0.60 (−1.83 to 0.62)	0.33				
Medication use			0.34				
No medication	1108 (53%)	REF					
Yes, not nephrotoxic	852 (41%)	−1.15 (−2.73 to 0.42)					
Yes, (potentially) nephrotoxic	136 (6%)	−0.14 (−3.38 to 3.09)					
Diabetes mellitus or cardiovascular disease^h^	317 (15%)	−5.17 (−8.88 to −1.45)	0.006	2.81 (−0.64 to 6.25)	0.11	2.15 (−1.43 to 5.72)	0.24
Hepatitis C virus RNA positive^i^	44 (2%)	0.67 (−3.06 to 4.40)	0.72				
Proteinuria (urine dipstick analysis)^j^			0.11				
Negative	1511 (72%)	REF					
Trace	159 (8%)	−1.62 (−3.31 to 0.06)	0.06				
Positive (≥+1)	22 (1%)	−2.62 (−6.68 to 1.43)	0.20				

AMPrEP, Amsterdam PrEP Demonstration Project; AUDIT, alcohol use disorder identification test; CI, confidence interval; CKD-EPI, Chronic Kidney Disease Epidemiology Collaboration; DUDIT, drug use disorder identification test; eGFR, estimated glomerular filtration rate; GEE, generalized estimating equations; PrEP, preexposure prophylaxis using tenofovir/emtricitabine; TFV-DP, intracellular tenofovir-diphosphate; 95% CI, 95% confidence interval.

a Baseline eGFR marked as ‘no PrEP’ since no PrEP was used.

b Measured at 12, 24, and 48 months.

c 193 missing.

d Indication of an alcohol use disorder.

e 187 missing.

f Indication of a drug use disorder.

g 200 missing.

h Deduced from reported medication used by participant.

i 13 missing.

j 404 missing (not measured at baseline; some missing at later visits).

**Figure 1:**
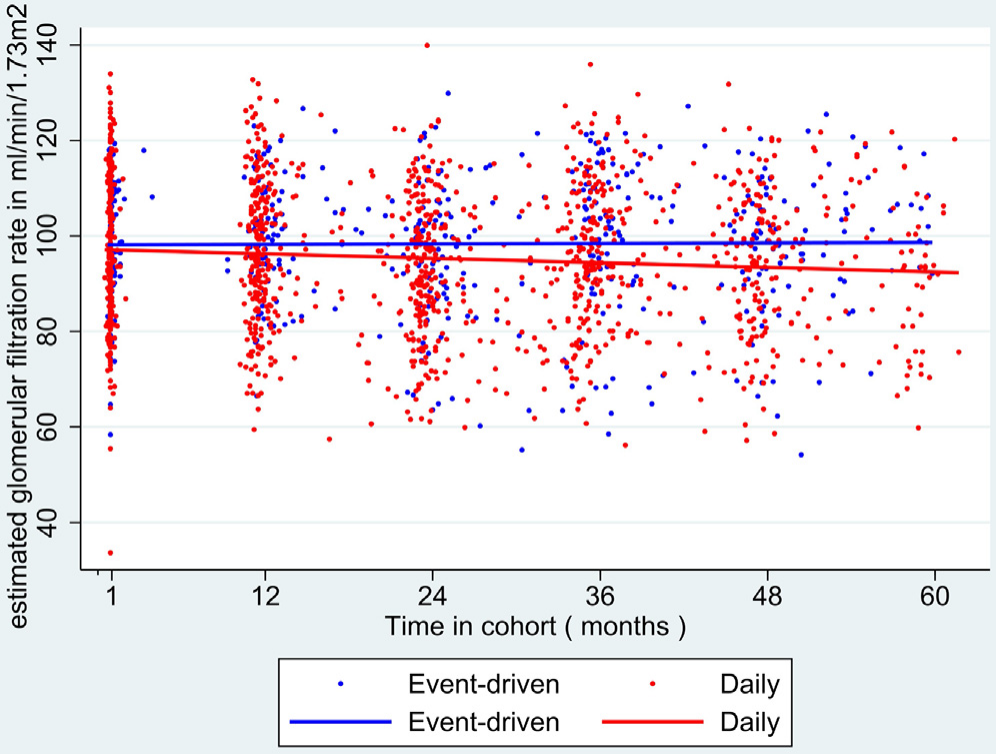
Scatterplot and regression lines of estimated glomerular filtration rate over time by PrEP regimen among AMPrEP participants, Amsterdam, The Netherlands, 2015-2020 (*N* = 351). AMPrEP, Amsterdam PrEP demonstration project; PrEP, preexposure prophylaxis.

**Figure 2:**
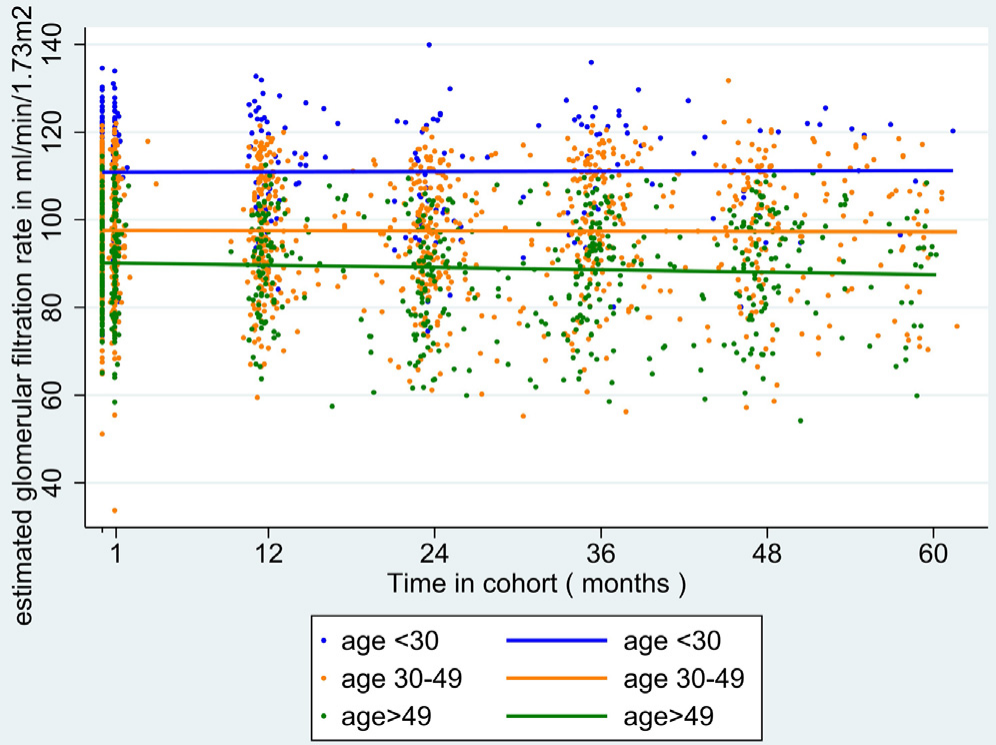
Scatterplot and regression lines of estimated glomerular filtration rate over time by age categories among AMPrEP participants, Amsterdam, The Netherlands, 2015–2020 (*N* = 351). AMPrEP, Amsterdam PrEP demonstration project; PrEP, preexposure prophylaxis.

When assessing change in eGFR over time, we noted a significant decline of 0.57 ml/min per 1.73 m^2^/yr (95% CI = − 1.06 to − 0.08, *P* = 0.02) in daily PrEP users, whereas no significant changes in event-driven PrEP users (+0.02 ml/min per 1.73 m^2^/yr, 95% CI = −0.83 to +0.79, *P* = 0.96, *P* for interaction = 0.30; Figure 1). In an analysis stratified by age category, no significant change in eGFR was observed in any age category, even for age ≥ 50 years [− 0.22 ml/min per 1.73 m^2^/yr [95% CI = − 0.89 to +0.44, *P* = 0.51, *P* for interaction = 0.71 (Figure 2)].

Sensitivity analysis of PrEP users with ≥ 5 years follow-up, never switching regimen, or eGFR calculated with the Cockcroft-Gault formula yielded qualitatively similar results (Figures S1 and S2 and Table S3).

### Proteinuria During PrEP Use

In univariable analysis, proteinuria was more common in those using daily PrEP, engaging in drug use during sex, and having diabetes or cardiovascular disease (Table 3). In multivariable analysis, proteinuria was more common in those using daily PrEP (adjusted odds ratio = 3.19, 95% CI = 1.31–7.76) and with diabetes or cardiovascular disease (adjusted odds ratio: 5.08, 95% CI = 1.92–13.45; Table 3). In sensitivity analysis including TFV-DP concentrations rather than PrEP regimen, TFV-DP concentrations were not associated with the odds of proteinuria (adjusted odds ratio = 1.00, 95% CI = 0.97–1.03 per 100 fmol/punch higher). Including only participants who never switched PrEP regimens yielded similar results (Figure S2).

**Table 3:** Determinants of proteinuria (urine dipstick analysis ≥ +1) in logistic regression among AMPrEP participants (*N* = 351, total number of proteinuria measurements = 5642), Amsterdam, The Netherlands 2015–2020

Characteristics	Number of measurements (%)	Univariable model		Multivariable model (including PrEP regimen [*n* = 5642])		Multivariable model (including TFV-DP concentration [*n* = 1179]^a^)	
		OR (95% CI)	*P*-value	aOR (95% CI)	*P*-value	aOR (95% CI)	*P*-value
Demographic characteristics							
Age at baseline (per 10 yrs older)		0.19 (−0.15 to 0.53)	0.26				
Self-declared ethnicity, not White	789 (14%)	0.51 (0.14–1.90)	0.32				
PrEP use							
Time of follow-up, per additional yr		0.85 (0.71–1.02)	0.80				
PrEP regimen, daily	4164 (74%)	2.98 (1.22–7.28)	0.02	3.19 (1.31–7.76)	0.01		
TFV-DP concentration (per 100 fmol/punch higher)^a^	1179	1.00 (0.97–1.04)	0.79			1.00 (0.97–1.03)	0.94
Variables that may influence kidney function							
AUDIT^b^^,^^c^							
Score ≥ 8 (indication)^d^	425 (8%)	1.78 (0.58–5.40)	0.31				
DUDIT^b^^,^^e^							
Score ≥ 8 (indication)^f^	567 (10%)	1.42 (0.53–3.82)	0.49				
Drug use during sex in the past 6 mo^b^^,^^g^	901 (16%)	3.12 (1.06–9.15)	0.04				
Medication use							
Yes, not nephrotoxic	2334 (41%)	1.54 (0.74–3.18)	0.25				
Yes, (potentially) nephrotoxic	351 (6%)	1.50 (0.35–6.46)	0.59				
Diabetes mellitus or cardiovascular disease^h^	759 (13%)	4.48 (1.73–11.61)	0.002	5.08 (1.92–13.45)	0.001	22.12 (2.52–194.48)	0.005
Hepatitis C virus RNA positive	105 (2%)	1.58 (0.37–6.80)	0.54				
eGFR (CKD-EPI 2021 equation, per 10 ml/min)^i^	5624	0.86 (0.69–1.09)	0.21				

AMPrEP, Amsterdam PrEP Demonstration Project; (a)OR, (adjusted) odds ratio; AUDIT, alcohol use disorder identification test; CKD-EPI, Chronic Kidney Disease Epidemiology Collaboration; DUDIT, drug use disorder identification test; eGFR, estimated glomerular filtration rate; PrEP, preexposure prophylaxis using tenofovir/emtricitabine; TFV-DP, intracellular tenofovir-diphosphate; 95% CI, 95% confidence interval.

a Measured at 12, 24, and 48 months.

b Measured yearly.

c 3844 missing.

d Indication of an alcohol use disorder.

e 3841 missing.

f Indication of a drug use disorder.

g 3843 missing.

h Deduced from reported medication use.

i Values carried forward from most recent visit with eGFR measurement.

### Participants With eGFR < 60 ml/min per 1.73 m^2^ or Persistent Proteinuria

Twelve participants (3.4%) had an eGFR < 60 ml/min per 1.73 m^2^, indicating moderate kidney impairment, at least once during follow-up (Table S4). Three participants (0.9%) had an eGFR < 60 ml/min per 1.73 m^2^ measured twice, although never at consecutive measurements. Eight participants (2.3%) had persistent proteinuria but never more than at 3 consecutive measurements (Table S5).

## Discussion

In this prospective, longitudinal, open-label demonstration project among 351 PrEP users in Amsterdam, we found that daily PrEP users had a slightly faster decline in eGFR over time and higher odds of developing proteinuria than event-driven PrEP users. Older people had lower mean eGFR values, most pronounced in people aged ≥ 50 years. In our study population, a decline in eGFR below 60 ml/min per 1.73 m^2^ never persisted and after adjustments for risk factors related to kidney disease, proteinuria during follow-up was not associated with tenofovir concentrations.

Our finding of a lower eGFR among daily PrEP users than event-driven PrEP users is consistent with other observational cohort studies.^18^, ^19^, ^20^, ^21^, ^22^ The ANRS-PREVENIR study showed a smaller eGFR decline for event-driven PrEP users than for daily PrEP users^22^; and in iPrEx-OLE, the participants had a higher eGFR when taking 2 pills or less per week compared with 7 or more pills per week.^18^ In addition to these studies, we found a significant association between TFV-DP concentration and eGFR, suggesting a dose-response relationship. Because TFV-DP remains longer in red blood cells (4–6 weeks) than TDF in plasma (several days),^23^ its quantification is more reflective of long-term adherence; this result is not biased by short-term adherence before study visits.

TDF is primarily associated with proximal tubular dysfunction.^24^ The lower eGFR that we observed in daily users may not indicate glomerular impairment, but rather an effect on the proximal renal tubules.^25^^,^^26^ Proteinuria may also reflect tubular dysfunction.^27^ We found that daily PrEP was associated with higher odds of proteinuria. Proteinuria was not independently associated with TFV-DP concentration.

Using the eGFR as a measure for kidney function has limitations. Differences in muscle mass can affect creatinine-based formulas, such as the Chronic Kidney Disease-Epidemiology Collaboration formula, causing an underestimation of the kidney function in more muscular people.^28^ Because the Cockcroft-Gault formula estimates creatinine clearance while correcting for body weight, we performed an additional analysis using this formula and observed similar effects of PrEP on the estimated creatinine clearance. To more accurately analyze the cumulative effect from treatment, the eGFR slope over time can be used.^29^ We observed a small but statistically significant decline in daily PrEP users, but not in event-driven PrEP users. Thus, there seems to be a small, though real, difference in eGFR between daily and event-driven PrEP users.

HIV infection is associated with renal impairment and being on certain antiretroviral agents is associated with a greater eGFR decline and risk of albuminuria.^30^ Our results indicate that the negative effects of preventive TDF use on eGFR and proteinuria are limited compared with effects of TDF use in people with HIV.

Declines to clinically significant levels of eGFR (< 60 ml/min per 1.73 m^2^) were only observed in 12 participants (3%), and none of those remained below 60 ml/min per 1.73 m^2^ during follow-up. In previous studies describing clinically significant declines in eGFR after TDF exposure, participants were people with HIV or chronic hepatitis B virus.^31^^,^^32^ A possible explanation for this discrepancy could be that TDF is more nephrotoxic in combination with other risk factors, such as HIV and hepatitis B virus infection, other antiretroviral medications, or comorbidities; a nephrotoxic effect does not emerge in people without these factors. For people with diabetes or cardiovascular disease using PrEP, we did not find a significantly lower eGFR. These comorbidities were, however, associated with proteinuria. Diabetes and cardiovascular disease are known to be associated with proteinuria reflecting glomerular damage and microvascular disease.^33^^,^^34^

The decline in eGFR over time seemed faster in people aged ≥ 50 years than in younger people, but it was not significant. This is in line with a previously reported meta-analysis.^8^ The observed decline among participants aged ≥ 50 years (− 0.22 ml/min per 1.73 m^2^/yr [*P* = 0.51]) was less than the reported normal range for aging (− 0.82 to − 1.15 ml/min per 1.73 m^2^/yr),^35^ and may not directly pose safety concerns for PrEP use in this age category. Our findings support the World Health Organization’s guidelines to make screening optional in individuals aged < 30 years, and optional or once around the time of initiation of PrEP in individuals aged ≤ 50 years without kidney-related comorbidities.^4^ Lower screening frequency of eGFR may be crucial in reducing costs and barriers for prescribing and using PrEP. Depending on available resources, it is worth considering systematically screening eGFR at least once at PrEP initiation.

An important strength of our study is the long follow-up duration. To our knowledge, this is the first study that assessed the effect of PrEP on renal outcomes over the long-term, with a median follow-up time of 54.2 months. Another strength is that we used the 2021 Chronic Kidney Disease-Epidemiology Collaboration formula to assess eGFR, which is a more accurate measure^36^ and represents kidney function more appropriately than other formulas, such as the Cockcroft-Gault formula. Moreover, we were able to assess associations between PrEP regimen and kidney function, and between TFV-DP concentration and kidney function, which indicate a likely dose-response relationship. We were also able to adjust for important potential confounders (i.e., comedication) and relevant comorbidities (i.e., diabetes and cardiovascular disease).

Nevertheless, this study has some limitations. First, the study population consisted mostly of White cisgender males with a median age of 41 years, which is not fully representative for the broader population that could benefit from PrEP. Second, information on diabetes and cardiovascular disease was deduced from comedication. The use of potentially nephrotoxic comedications was low, so statistical power was limited to assess associations. Third, we could not correct for smoking, HbA1c, body mass index, and lean body weight because data on these measures were not collected. Fourth, for measurement of proteinuria, we used urine dipstick analysis with manual reading, where quantifying is less accurate.^17^ Cystatin C-based eGFR was also not assessed. In contrast to primary care and hospital settings, for the purpose of screening in low CKD risk populations of a public health setting, dipstick analysis and creatinine-based eGFR are generally acceptable methods.^37^ Lastly, following oral PrEP guidelines, we excluded participants with a baseline eGFR < 60 ml/min per 1.73 m^2^. People with decreased kidney function who are vulnerable to HIV require a tailored approach, using more frequent kidney function monitoring during PrEP follow-up or other PrEP modalities with a better renal safety profile, such as tenofoviralafenamide^38^ or cabotegravir.^39^

In conclusion, we found that PrEP use did not coincide with clinically significant lower eGFR or proteinuria over the course of 5 years of PrEP use. However, daily PrEP users had a slightly lower eGFR and higher odds of proteinuria than event-driven users. The decline in eGFR over time seemed faster in people aged ≥ 50 years than in younger people, albeit the effect was not significant and within normal range for ageing. Based on these results, optional or 1-time kidney function screening for PrEP users younger than 50 years without comorbidities related to chronic kidney dysfunction is appropriate. Considering that frequent renal monitoring among PrEP users involves extra costs and may increase barriers in prescribing and using PrEP, our data support reduced kidney function monitoring in people at low risk for kidney dysfunction.

## Appendix

### List of the members of the Amsterdam PrEP Project Team in the HIV Transmission Elimination AMsterdam Initiative (H-TEAM)

H-TEAM members: T. van Benthem^1^, D. Bons^2^, G.J. de Bree^3,4^, P. Brokx^5^, U. Davidovich^1,6^, S. Hendriks^7^, S.E. Geerlings^4^, M. Heidenrijk^3^, E. Hoornenborg^1^, J. Heijne^1,4^, P. Reiss^3,5^, A. van Sighem^8^, M. van der Valk^4,8^, J. de Wit^9^, P. Zantkuijl^7^.

H-TEAM Project Management: N. Schat^3^, L. Dol^3^.

H-TEAM additional collaborators: M. van Agtmael^10^, J. Ananworanich^11^, D. Van de Beek^12^, G.E.L. van den Berk^13^, D. Bezemer^8^, A. van Bijnen^7^, J.P. Bil^1^, W.L. Blok^12^, S. Bogers^4^, M. Bomers^10^, A. Boyd^1,8^, W. Brokking^14^, D. Burger^15^, K. Brinkman^13^, N. Brinkman^13^, M. de Bruin^16^, S. Bruisten^1^, L. Coyer^1^, R. van Crevel^17^, M. Dijkstra^1^, Y.T. van Duijnhoven^1^, A. van Eeden^14^, L. Elsenburg^14^, M.A.M. van den Elshout^1^, E. Ersan^18^, P. E.V. Felipa^1^, T.B.H. Geijtenbeek^19^, J. van Gool^1^, A. Goorhuis^4^, M. Groot^14^, C.A. Hankins^3^, A. Heijnen^20,21^, M.M.J Hillebregt^8^, M. Hommenga^1^, J.W. Hovius^4^, Y. Janssen^22^, K. de Jong^1^, V. Jongen^1^, N.A. Kootstra^23^, R.A. Koup^24^, F.P. Kroon^25^, T.J.W. van de Laar^26,27^, F. Lauw^28^, M. M. van Leeuwen^5^, K. Lettinga^29^, I. Linde^1^, D.S.E. Loomans^1^, I.M. van der Lubben^1^, J.T. van der Meer^4^, T. Mouhebati^7^, B.J. Mulder^1^, J. Mulder^30^, F.J. Nellen^4^, A. Nijsters^7^, H. Nobel^4^, E.L.M. Op de Coul^31^, E. Peters^10^, I.S. Peters^1^, T. van der Poll^4^, O. Ratmann^32^, C. Rokx^33^, M.F. Schim van der Loeff^1,34^, W.E.M. Schoute^13^, J. Schouten^1^, J. Veenstra^29^, A. Verbon^33^, F. Verdult^5^, J. de Vocht^10^, H.J. de Vries^1,34,35^, S. Vrouenraets^29^, M. van Vugt^4^, W.J. Wiersinga^4^, F.W. Wit^4,6^, L.R. Woittiez^4^, S. Zaheri^8^, P. Zantkuijl^7^, Żakowicz^36^, M.C. van Zelm^37^, H.M.L. Zimmermann^1^

^1^ Department of Infectious Diseases, Public Health Service of Amsterdam, Amsterdam, the Netherlands

^2^ Trans United Europe, Amsterdam, The Netherlands

^3^ Department of Global Health, Amsterdam UMC – location AMC, and Amsterdam Institute for Global Health and Development, Amsterdam, The Netherlands

^4^ Department of Internal Medicine, Division of Infectious Diseases, Amsterdam UMC – location AMC, Amsterdam, The Netherlands

^5^ Dutch Association of PLHIV, Amsterdam, The Netherlands

^6^ Department of Social Psychology, University of Amsterdam, Amsterdam, The Netherlands

^7^ Soa Aids Nederland, Amsterdam, The Netherlands

^8^ Stichting HIV Monitoring, Amsterdam, The Netherlands

^9^ Department of Interdisciplinary Social Science: Public Health, Utrecht University, Utrecht, The Netherlands

^10^ Department of Internal Medicine, Amsterdam UMC – location VUMC, Amsterdam, The Netherlands

^11^ US Military HIV Research Program and the Henry M. Jackson Foundation for the Advancement of Military Medicine, Bethesda, United States

^12^ Center of Infection and Immunity Amsterdam (CINIMA), Department of Neurology, Amsterdam UMC – location AMC, Amsterdam, The Netherlands

^13^ Department of internal medicine, OLVG – location East, Amsterdam, The Netherlands

^14^ DC Klinieken, Amsterdam, The Netherlands

^15^ Department of Pharmacy, Radboud University Nijmegen Medical Center, Nijmegen, The Netherlands

^16^ Aberdeen Health Psychology Group, Institute of Applied Health Sciences, University of Aberdeen, Aberdeen, United Kingdom

^17^ Department of Internal Medicine, Radboud University Nijmegen Medical Center, Nijmegen, The Netherlands

^18^ Department of General Practice, Amsterdam UMC – location AMC, University of Amsterdam, Amsterdam, The Netherlands

^19^ Laboratory of Experimental Immunology, Amsterdam UMC – location AMC Amsterdam, The Netherlands

^20^ Sexology Center Amsterdam, Amsterdam, The Netherlands

^21^ GP practice Heijnen & de Meij, Amsterdam, The Netherlands

^22^ Primary Care Amsterdam and Almere (Elaa), Amsterdam, The Netherlands

^23^ Laboratory for Viral Immune Pathogenesis, Amsterdam UMC – location AMC Amsterdam, The Netherlands

^24^ Immunology Laboratory, Vaccine Research Center, National Institute of Allergy and Infectious Diseases, National Institutes of Health, Rockville, Maryland, USA

^25^ Department of Infectious Diseases, Leiden University Medical Center, Leiden, The Netherlands

^26^ Department of Medical Microbiology, OLVG, Amsterdam, The Netherlands

^27^ Department of Donor Medicine Research, Laboratory of Blood-borne Infections, Sanquin Research, Amsterdam, The Netherlands

^28^ Department of Internal Medicine, Medical Center Jan van Goyen, Amsterdam, The Netherlands

^29^ Department of Internal Medicine, OLVG – location West, Amsterdam, The Netherlands

^30^ Department of Internal Medicine, Slotervaart Hospital (former), Amsterdam, The Netherlands

^31^ Epidemiology and Surveillance Unit, Center for Infectious Disease Control, National Institute of Public Health and the Environment, The Netherlands

^32^ School of Public Health, Faculty of Medicine, Imperial College London, London, United Kingdom

^33^ Department of Internal Medicine and Infectious Diseases, Erasmus Medical Center, Rotterdam, The Netherlands

^34^ Center for Infection and Immunology, Amsterdam (CINIMA), Amsterdam UMC – location AMC, University of Amsterdam, Amsterdam, The Netherlands

^35^ Department of Dermatology, Amsterdam UMC – location AMC, University of Amsterdam, Amsterdam, The Netherlands

^36^ AIDS Healthcare Foundation, Amsterdam, The Netherlands

^37^ Department of Virology, Erasmus Medical Center, Rotterdam, The Netherlands

## Disclosure

AB received speaker fees from Gilead Sciences. EH received an unrestricted research grant from Gilead Sciences, paid to their institution. HV received fees from AbbVie and Abbott, paid to their institution. MSvdL received a grant from GSK and fees from NOVOSANIS and MSD, all paid to their institution. All the other authors declared no competing interests.

## Supplementary Material

Supplementary File (PDF) **Figure S1.** Scatterplot and regression lines of estimated glomerular filtration rate over time by PrEP regimen among AMPrEP participants who completed 5 or more years of follow-up, Amsterdam (*n* = 54). **Figure S2.** Scatterplot and regression lines of estimated glomerular filtration rate over time by PrEP regimen among AMPrEP participants who never switched PrEP regimen, Amsterdam (*n* = 201). **Figure S3.** Stacked bar graph of proteinuria measured by urine dipstick analysis over time (cumulative percentages) among AMPrEP participants, Amsterdam, The Netherlands, 2015–2020. **Table S1.** Baseline characteristics measured at study enrolment (2015–2016), and follow-up time (2015–2020) of AMPrEP participants included and excluded from analysis, Amsterdam, The Netherlands. **Table S2.** Baseline characteristics measured at study enrolment (2015–2016), and follow-up time (2015–2020) of AMPrEP participants by baseline eGFR (using the CKD-EPI 2021 equation), Amsterdam, The Netherlands. **Table S3.** Determinants of eGFR (using the Cockroft-Gault equation) in linear regression using GEE among AMPrEP participants (*n* = 351, total number of creatinine measurements = 2096), Amsterdam, The Netherlands, 2015–2020. **Table S4.** Descriptives of AMPrEP study participants whose eGFR (using the CKD-EPI 2021 equation) dropped below 60 ml/min per 1.73 m^2^ (*n* = 12), Amsterdam, The Netherlands, 2015–2020. **Table S5.** Descriptives of participants with persistent proteinuria, defined as 2 or more consecutive study visits with ≥ 1+ proteinuria (*n* = 8) among AMPrEP study participants, Amsterdam, The Netherlands, 2015–2020. STROBE Checklist.

## Footnotes

Supplementary File (PDF)
**Figure S1.** Scatterplot and regression lines of estimated glomerular filtration rate over time by PrEP regimen among AMPrEP participants who completed 5 or more years of follow-up, Amsterdam (*n* = 54). **Figure S2.** Scatterplot and regression lines of estimated glomerular filtration rate over time by PrEP regimen among AMPrEP participants who never switched PrEP regimen, Amsterdam (*n* = 201). **Figure S3.** Stacked bar graph of proteinuria measured by urine dipstick analysis over time (cumulative percentages) among AMPrEP participants, Amsterdam, The Netherlands, 2015–2020. **Table S1.** Baseline characteristics measured at study enrolment (2015–2016), and follow-up time (2015–2020) of AMPrEP participants included and excluded from analysis, Amsterdam, The Netherlands. **Table S2.** Baseline characteristics measured at study enrolment (2015–2016), and follow-up time (2015–2020) of AMPrEP participants by baseline eGFR (using the CKD-EPI 2021 equation), Amsterdam, The Netherlands. **Table S3.** Determinants of eGFR (using the Cockroft-Gault equation) in linear regression using GEE among AMPrEP participants (*n* = 351, total number of creatinine measurements = 2096), Amsterdam, The Netherlands, 2015–2020. **Table S4.** Descriptives of AMPrEP study participants whose eGFR (using the CKD-EPI 2021 equation) dropped below 60 ml/min per 1.73 m^2^ (*n* = 12), Amsterdam, The Netherlands, 2015–2020. **Table S5.** Descriptives of participants with persistent proteinuria, defined as 2 or more consecutive study visits with ≥ 1+ proteinuria (*n* = 8) among AMPrEP study participants, Amsterdam, The Netherlands, 2015–2020. STROBE Checklist.
