# Supplementary material for: Renal Outcomes Over the Course of 5 Years of Oral HIV Preexposure Prophylaxis Using Tenofovir Disoproxil/Emtricitabine
Source: Kidney Int Rep. 2025 Mar 25;10(6):2003–14. doi: 10.1016/j.ekir.2025.03.033 (PMC12231006; doi:10.1016/j.ekir.2025.03.033)
Supplement: Supplementary File (PDF) — Figure S1. Scatterplot and regression lines of estimated glomerular filtration rate over time by PrEP regimen among AMPrEP participants who completed 5 or more years of follow-up, Amsterdam (n = 54). Figure S2. Scatterplot and regression lines of estimated glomerular filtration rate over time by PrEP regimen among AMPrEP participants who never switched PrEP regimen, Amsterdam (n = 201). Figure S3. Stacked bar graph of proteinuria measured by urine dipstick analysis over time (cumulative percentages) among AMPrEP participants, Amsterdam, The Netherlands, 2015–2020. Table S1. Baseline characteristics measured at study enrolment (2015–2016), and follow-up time (2015–2020) of AMPrEP participants included and excluded from analysis, Amsterdam, The Netherlands. Table S2. Baseline characteristics measured at study enrolment (2015–2016), and follow-up time (2015–2020) of AMPrEP participants by baseline eGFR (using the CKD-EPI 2021 equation), Amsterdam, The Netherlands. Table S3. Determinants of eGFR (using the Cockroft-Gault equation) in linear regression using GEE among AMPrEP participants (n = 351, total number of creatinine measurements = 2096), Amsterdam, The Netherlands, 2015–2020. Table S4. Descriptives of AMPrEP study participants whose eGFR (using the CKD-EPI 2021 equation) dropped below 60 ml/min per 1.73 m2 (n = 12), Amsterdam, The Netherlands, 2015–2020. Table S5. Descriptives of participants with persistent proteinuria, defined as 2 or more consecutive study visits with ≥ 1+ proteinuria (n = 8) among AMPrEP study participants, Amsterdam, The Netherlands, 2015–2020. STROBE Checklist. [file mmc1.pdf]

Supplementary materials to

Renal outcomes over the course of five years of oral HIV pre-exposure prophylaxis using tenofovir disoproxil fumarate/ emtricitabine among men who have sex with men

Dita C. BOLLUYT*[1], Mark A.M. VAN DEN ELSHOUT*[1], Eline S. WIJSTMA[1], Anders BOYD[1,2,3,5], Elske HOORNENBORG[1], Henry JC DE VRIES[1,3,4], Maria PRINS[1,3,5], Liffert VOGT[6], Maarten F. SCHIM VAN DER LOEFF[1,3,5], on behalf of the Amsterdam PrEP Project team in the HIV Transmission Elimination AMsterdam Initiative (H-TEAM)
*authors contributed equally

Authors' affiliations

[1] Department of Infectious Diseases, Public Health Service of Amsterdam, Amsterdam, the Netherlands

[2] HIV Monitoring Foundation, Amsterdam, the Netherlands

[3] Amsterdam institute for Immunology & Infectious Diseases (AII), Amsterdam UMC, University of Amsterdam, Amsterdam, the Netherlands

[4] Amsterdam UMC location University of Amsterdam, Department of Dermatology, Amsterdam, the Netherlands.

[5] Department of Infectious Diseases, Amsterdam UMC, University of Amsterdam, Amsterdam, the Netherlands.

[6] Department of Nephrology, Amsterdam UMC, University of Amsterdam, Amsterdam, the Netherlands.

Table of contents

Supplementary Table S1: Baseline characteristics measured at study enrolment (2015-2016), and follow-up time (2015-2020) of AMPrEP participants included and excluded from analysis, Amsterdam, The Netherlands 2

Supplementary Table S2: Baseline characteristics measured at study enrolment (2015-2016), and follow-up time (2015-2020) of AMPrEP participants by baseline eGFR (using the CKD-EPI 2021 equation), Amsterdam, The Netherlands 4

Figure S1. Scatterplot and regression lines of estimated glomerular filtration rate over time by PrEP regimen among AMPrEP participants who completed five or more years of follow-up, Amsterdam (n=54). the Netherlands, 2015-2020 (n=54). 6

Figure S2. Scatterplot and regression lines of estimated glomerular filtration rate over time by PrEP regimen among AMPrEP participants who who never switched PrEP regimen, Amsterdam (n=201), the Netherlands, 2015-2020 (n=201). 6

Figure S3. Stacked bar graph of proteinuria measured by urine dipstick analysis over time (cumulative percentages) among AMPrEP participants, Amsterdam, the Netherlands, 2015-2020. Proteinuria was measured at 1 month and every measured every three months thereafter (n= 5,642) 7

Supplementary Table S2: Determinants of eGFR (using the Cockroft-Gault equation[a]) in linear regression using GEE among AMPrEP participants (n=351, total number of creatinine measurements=2,096), Amsterdam, the Netherlands, 2015-2020 8

Supplementary Table S3: Descriptives of AMPrEP study participants whose eGFR (using the CKD-EPI 2021 equation) dropped below 60 mL/min/1.73 m^2 (n=12), Amsterdam, the Netherlands, 2015-2020 9

Supplementary Table S4: Descriptives of participants with persistent proteinuria, defined as two or more consecutive study visits with ≥1+ proteinuria (n=8) among AMPrEP study participants, Amsterdam, the Netherlands, 2015-2020 10

STROBE Statement—checklist of items that should be included in reports of observational studies 11

Supplementary Table S1: Baseline characteristics measured at study enrolment (2015-2016), and follow-up time (2015-2020) of AMPrEP participants included and excluded from analysis, Amsterdam, The Netherlands

	Total included (N=351)		Excluded (N=25)		
	N	%[a]	N	%[a]	p-value
**Demographic characteristics**					
PrEP regimen					0.54
Daily	255	73%	18	72%	
Event-driven	96	27%	7	28%	
Age (years)					**0.02**
Median (IQR)	41	(33-49)	36	(29-41)	
Age (years, categorized)					0.23
≤30	63	18%	7	28%	
30 to 39	105	30%	10	40%	
40 to 49	96	27%	5	20%	
≥50	87	25%	3	12%	
Gender identity					0.81
Male	349	99%	25	100%	
Transgender woman	2	1%	0	0%	
Self-declared ethnicity					0.11
Arabic	1	0%	2	8%	
Asian	10	3%	0	0%	
Black African	6	2%	0	0%	
Black Other	5	1%	0	0%	
Mixed	24	7%	2	8%	
White	300	85%	20	83%	
Other	5	1%	0	0%	
Education level					**0.05**
No university / university for applied sciences	80	23%	10	40%	
University / university for applied sciences	271	77%	15	60%	
Employment					0.35
Employed	270	78%	20	80%	
Unemployed	17	5%	2	8%	
Other (retired, volunteer, disabled, student)	60	17%	3	12%	
Net monthly income in Euro's					0.66
≤1700	92	27%	7	33%	
1701-2950	144	43%	7	33%	
>2950	100	30%	7	33%	
**Variables that may influence renal function**					
Alcohol use disorder identification test (AUDIT)					0.06
Score <8 (no indication)[b]	254	73%	12	55%	
Score ≥8 (indication)[c]	94	27%	10	45%	
Drug use disorder identification test (DUDIT)					0.53
Score <8 (no indication)[d]	223	64%	13	62%	
Score ≥8 (indication)[e]	128	36%	8	38%	
Drug use during sex in the past 6 months					0.27
No	159	45%	9	38%	
Yes	184	54%	15	63%	
Current medication use					0.83
No	207	59%	17	68%	
Yes, not-nephrotoxic	124	35%	7	28%	
Yes, (potentially) nephrotoxic	20	6%	1	4%	

Diabetes mellitus[f]	8	2%	0	0%	
Cardiovascular disease[f]	48	14%	1	4%	
Creatinine (µmol/L)					0.60
Mean (SD)	86	(12)	87	(15)	
eGFR CKD-EPI, mL/min/1.73 m^2					0.45
Mean (SD)	100	(14)	102	(16)	
KDIGO kidney function stage					
Normal ≥90 mL/min/1.73 m^2	261	74%	20	80%	
Mild impairment 60-89 mL/min/1.73 m^2	89	25%	5	20%	
Moderate impairment 30-59 mL/min/1.73 m^2	1	0.3%	0	0%	
Proteinuria, dipstick[g]					0.76
Negative	283	84%	12	80%	
Trace	50	15%	3	20%	
≥+1	4	1%	0	0%	
HCV status					0.59
RNA negative	335	96%	24	96%	
RNA positive	14	4%	1	4%	
**Follow-up**					
Time of follow-up in months					
Median (IQR)	54	(47-58)	4	(1-10)	

AMPrEP: Amsterdam PrEP demonstration project; CKD-EPI: Chronic Kidney Disease Epidemiology Collaboration; eGFR: estimated glomerular filtration rate (using the CKD-EPI 2021 equation); HCV: hepatitis C virus; IQR: interquartile range; KDIGO: Kidney Disease Improving Global Outcomes Group; SD: standard deviation; PrEP: pre-exposure prophylaxis.

p-values for continuous variables calculated with t-test or Mann Whitney U-test, for categorical variables with Pearson's Chi-squared test or a Fisher's exact test (n<5). [a]Percentages may not total 100 due to rounding; [b]No indication of an alcohol use disorder; [c]Indication of an alcohol use disorder; [d]No indication of a drug use disorder; [e]Indication of a drug use disorder; [f]deduced from reported medication use; [g]measured at first study visit (one month after enrolment).

Supplementary Table S2: Baseline characteristics measured at study enrolment (2015-2016), and follow-up time (2015-2020) of AMPrEP participants by baseline eGFR (using the CKD-EPI 2021 equation), Amsterdam, The Netherlands

	Total (N=351)		eGFR ≥90 mL/min/1.73 m^2 (N=261)		eGFR <90 mL/min/1.73 m^2 (N=90)		
	N	%[a]	N	%[a]	N	%[a]	p-value
**Demographic characteristics**							
PrEP regimen							0.92
Daily	255	73%	190	73%	65	72%	
Event-driven	96	27%	71	27%	25	27%	
Age (years)							**<0.001**
Median (IQR)	41	(33-49)	38	(31-46)	47	(39-55)	
Age (years, categorized)							**<0.001**
<30	63	18%	60	23%	3	3%	
30 to 39	105	30%	82	31%	23	26%	
40 to 49	96	27%	69	26%	27	30%	
≥50	87	25%	50	19%	37	41%	
Gender identity							0.52
Male	349	99%	259	99%	90	100%	
Transgender woman	2	1%	2	1%	0	0%	
Self-declared ethnicity							0.37
Arabic	1	0%	0	0%	1	1%	
Asian	10	3%	9	4%	1	1%	
Black African	6	2%	6	2%	0	0%	
Black Other	5	1%	3	1%	2	2%	
Mixed	24	7%	18	7%	6	7%	
white	300	85%	221	85%	79	88%	
Other	5	1%	4	2%	1	1%	
Education level							0.36
No university/university for applied sciences	80	23%	63	24%	17	19%	
University/university for applied sciences	271	77%	198	76%	73	81%	
Employment[b]							**0.02**
Employed	270	78%	195	75%	75	85%	
Unemployed	17	5%	14	5%	3	3%	
Other (retired, volunteer, disabled, student)	60	17%	50	19%	10	11%	
Net monthly income in Euro's[c]							**0.001**
≤1700	92	27%	81	32%	11	13%	
1701-2950	144	43%	106	42%	38	45%	
>2950	100	30%	64	26%	36	42%	
**Variables that may influence kidney function**							
Alcohol use disorder identification test (AUDIT)[d]							0.40
Score <8 (no indication)[g]	254	73%	186	72%	68	76%	
Score ≥8 (indication)[h]	94	27%	73	28%	21	24%	
Drug use disorder identification test (DUDIT)							0.58
Score <8 (no indication)[i]	223	64%	168	64%	55	61%	
Score ≥8 (indication)[k]	128	36%	93	36%	35	39%	
Drug use during sex in the past 6 months[e]							0.12
No	159	45%	125	49%	34	39%	
Yes	184	54%	131	51%	53	61%	
Current medication use							0.32

No	207	59%	160	61%	47	53%	
Yes, not-nephrotoxic	124	35%	89	34%	35	39%	
Yes, (potentially) nephrotoxic	20	6%	13	5%	7	8%	
Diabetes mellitus[m]	8	2%	7	3%	1	1%	0.35
Cardiovascular disease[m]	48	14%	31	12%	17	19%	0.09
Weight (kg)							
Total mean (SD)	82	(14)	81	(15)	83	(12)	0.21
among participants with CVD or diabetes	89	(15)					**0.0001**[p]
among other participants	81	(13)					
Creatinine (μmol/L)							
Mean (SD)	86	(12)	81	(9)	100	(11)	
eGFR CKD-EPI 2021, mL/min/1.73 m^2							
Mean (SD)	100	(14)	106	(10)	81	(7)	
KDIGO kidney function stage							
Normal ≥90 mL/min/1.73 m^2	261	74%					
Mild impairment 60-89 mL/min/1.73 m^2	89	25%					
Moderate impairment 30-59 mL/min/1.73 m^2	1	0,3%					
Proteinuria, dipstick[c, o]							0.1
Negative	283	84%	198	86%	81	80%	
Trace	50	15%	31	13%	17	17%	
≥+1	4	1%	1	0.4%	4	3%	
HCV status[f]							0.49
HCV-RNA positive	335	96%	250	96%	85	96%	
HCV-RNA negative	14	4%	10	4%	4	4%	
**Follow-up**							
Time of follow-up in months							0.11
Median (IQR)	54	(47-58)	54	(46-57)	55	(49-58)	

AMPrEP: Amsterdam PrEP demonstration project; CKD-EPI: Chronic Kidney Disease Epidemiology Collaboration; CVD: cardiovascular disease; eGFR: estimated glomerular filtration rate (using the CKD-EPI 2021 equation); HCV: hepatitis C virus; IQR: interquartile range; KDIGO: Kidney Disease Improving Global Outcomes Group; SD: standard deviation; PrEP: pre-exposure prophylaxis.

p-values for continuous variables calculated with t-test or Mann Whitney U-test, for categorical variables with Pearson's Chi-squared test or a Fisher's exact test (n<5). [a]Percentages may not total 100 due to rounding; [b]4 missing; [c]15 missing; [d]3 missing; [e]8 missing; [f]2 missing; [g]No indication of an alcohol use disorder; [h]Indication of an alcohol use disorder; [i]No indication of a drug use disorder; [k]Indication of a drug use disorder; [m]deduced from medication use; [o]measured at first study visit (one month after enrolment); [p]comparing weight between participants with and without CVD or diabetes.

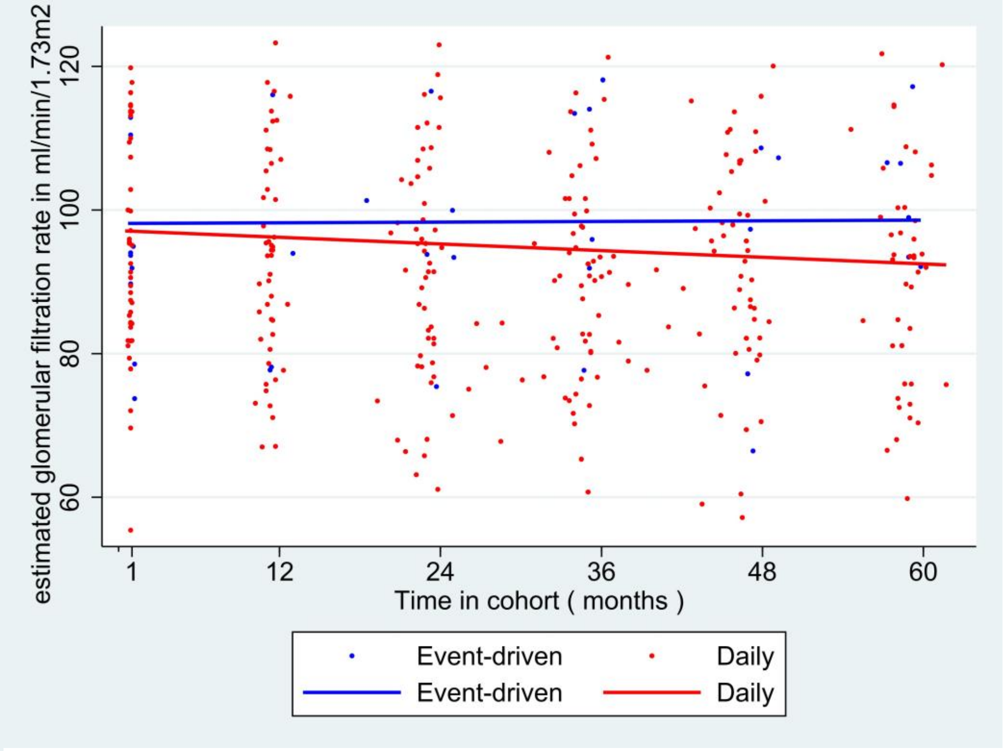

Figure S1. Scatterplot and regression lines of estimated glomerular filtration rate over time by PrEP regimen among AMPrEP participants who completed five or more years of follow-up, Amsterdam (n=54).

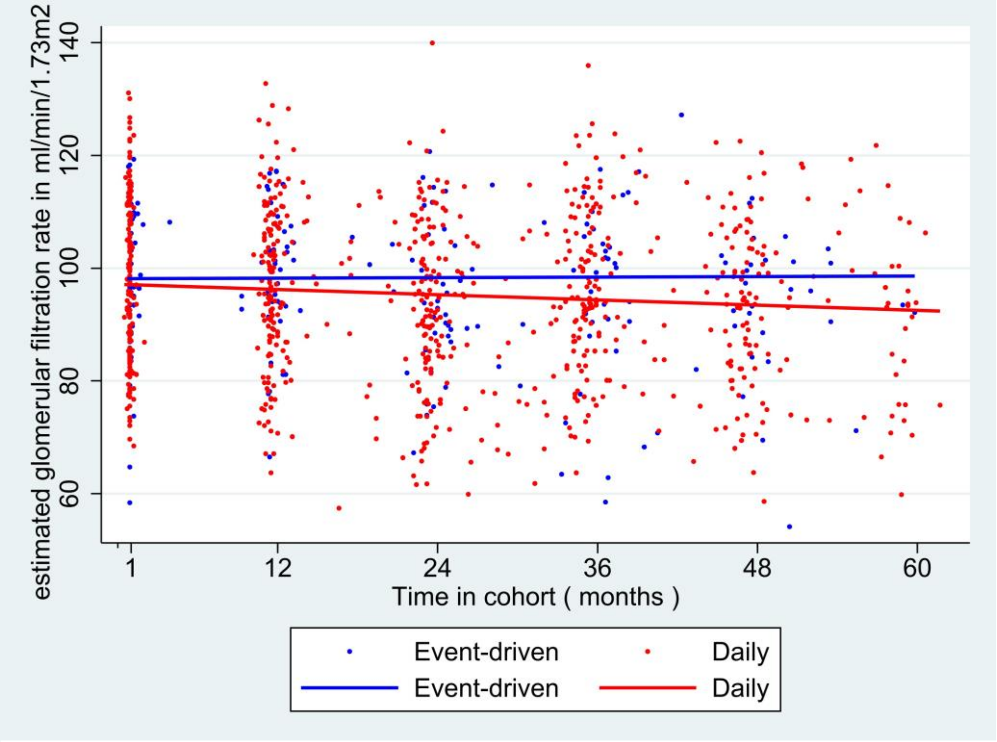

Figure S2. Scatterplot and regression lines of estimated glomerular filtration rate over time by PrEP regimen among AMPrEP participants who who never switched PrEP regimen, Amsterdam (n=201),

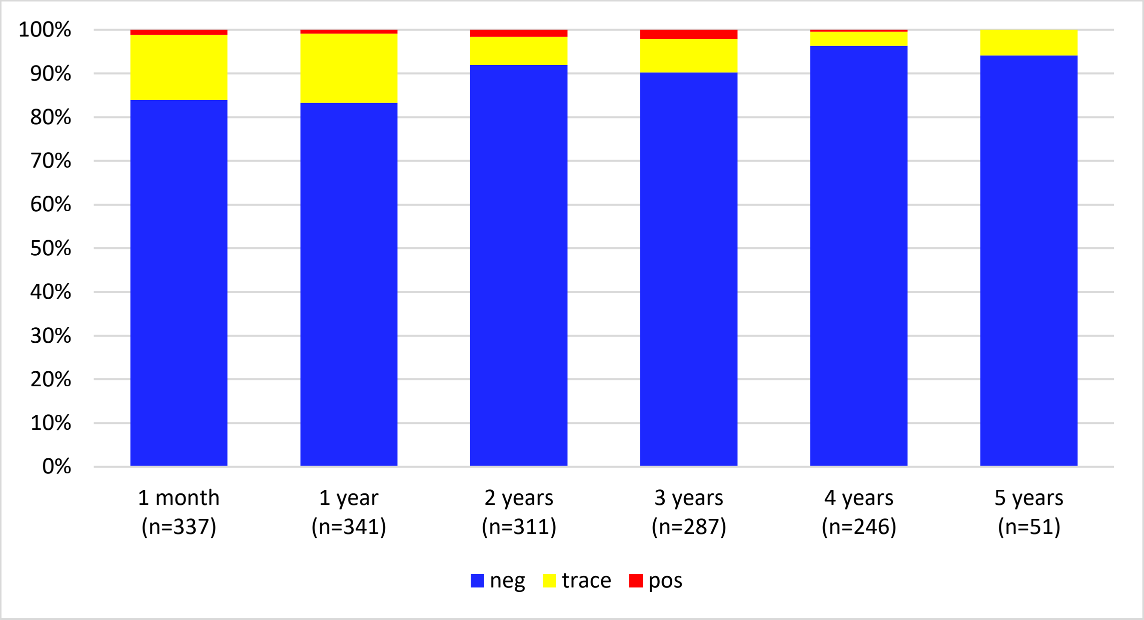

Figure S3. Stacked bar graph of proteinuria measured by urine dipstick analysis over time (cumulative percentages) among AMPrEP participants, Amsterdam, the Netherlands, 2015-2020.

Supplementary Table S3: Determinants of eGFR (using the Cockroft-Gault equation[a]) in linear regression using GEE among AMPrEP participants (n=351, total number of creatinine measurements=2,096), Amsterdam, the Netherlands, 2015-2020

		Univariable model		Multivariable model (including PrEP regimen (n=2,093))		Multivariable model (including TFV-DP concentration (n=832)[a])	
	**Number of measurements (%)**	**difference in eGFR in mL/min/1.73 m²(95% CI)**	**P-value**	**difference in eGFR in mL/min/1.73 m² (95% CI)**	**P-value**	**difference in eGFR in mL/min/1.73 m² (95% CI)**	**P-value**
**Demographic characteristics**							
Age at baseline (per 10 years older)		-7.68 (-9.66 to 5.70)	<0.0001	-9.08 (-11.17 to -6.98)	<0.0001	-8.94 (-11.05 to -6.83)	<0.0001
Self-declared ethnicity, not-white	290 (14%)	2.01 (-5.81 to 9.83)	0.61				
**PrEP use**							
Time of follow-up, per additional year		-0.75 (-1.10 to -0.40)	<0.0001	-0.40 (-0.78 to -0.02)	0.04	-1.21 (-2.14 to -0.28)	0.01
PrEP regimen[b]							
Daily	1,273 (61%)	-4.44 (-5.53 to -3.35)	<0.0001	-3.73 (-4.82 to -2.63)	<0.0001		
Event-driven	472 (23%)	-1.98 (-3.79 to -0.16)	0.03	-1.03 (-2.93 to 0.88)	0.29		
TFV-DP concentration (per 100 fmol/punch higher)[c]	832	-0.12 (-0.21 to 0.03)	0.01			-0.28 (-0.44 to -0.11)	0.001
**Variables that may influence kidney function**							
Alcohol use disorder identification test (AUDIT)[d]							
Score ≥8 (indication)[k]	478 (23%)	-0.08 (-2.20 to 2.04)	0.94				
Drug use disorder identification test (DUDIT)[e]							
Score ≥8 (indication)[l]	625 (30%)	-1.02 (-0.52 to 2.55)	0.20				
Drug use during sex in the past 6 months[f]	965 (46%)	-1.12 (-2.71 to 0.47)	0.17				
Medication use			0.34				
No medication	1,108 (83%)	REF					
Yes, not-nephrotoxic	852 (41%)	-1.37 (-3.33 to 0.60)	0.17				
Yes, (potentially) nephrotoxic	136 (6%)	-0.10 (-4.25 to 4.06)	0.96				
Diabetes mellitus or cardiovascular disease[m]	317 (15%)	-0.52 (-8.68 to 7.64)	0.90	11.37 (3.76 to 18.98)	0.003	10.47 (2.82 to 18.14)	0.007
Hepatitis C virus RNA positive[g]	44 (2%)	1.89 (-2.20 to 5.98)	0.37				
Proteinuria (urine dipstick analysis)[h]			0.20				
Negative	1,511 (72%)	REF					
Trace	159 (8%)	-1.69 (-3.79 to 0.40)	0.11				
Positive (≥+1)	22 (1%)	-3.09 (-8.20 to 2.02)	0.24				

AMPrEP: Amsterdam PrEP Demonstration Project; eGFR = estimated glomerular filtration rate; GEE = generalized estimating equations; PrEP = pre-exposure prophylaxis; TFV-DP = intracellular tenofovir-diphosphate; 95%CI: 95% confidence interval; [a]weight was only measured at baseline; [b]baseline eGFR marked as 'no PrEP' since no PrEP was used; [c]measured at 12, 24 and 48 months; [d]193 missing; [e]187 missing; [f]200 missing; [g]13 missing; [h]404 missing (not measured at baseline, and some at later visits); [k]indication of an alcohol use disorder; [l]indication of a drug use disorder; [m]deduced from reported medication use by participant.

Supplementary Table S4: Descriptives of AMPrEP study participants whose eGFR (using the CKD-EPI 2021 equation) dropped below 60 mL/min/1.73 m^2 (n=12), Amsterdam, the Netherlands, 2015-2020

	**P1**	**P2**	**P3**	**P4**	**P5**	**P5;2nd**	**P6**	**P7**	**P7;2nd**	**P8**	**P9**	**P10**	**P11**	**P11;2nd**	**P12**		**Total**
**Demographic characteristics**																	
Age at baseline (years)	35	37	37	39	40	40	47	47	47	48	50	64	67	67	71	*mean*	**46.5**
Self-reported ethnicity	black	?	white	white	white	white	white	white	white	white	white	white	white	white	white		
**eGFR measurements**																	
eGFR at baseline (mL/min/1.73 m^2)	94.8	51.1	94.8	72.5	88.5	88.5	67.5	64.8	64.8	82.1	72.2	79.2	72.1	72.1	93,4	*mean*	**77.8**
eGFR value when <60 mL/min/1.73 m^2	58.6	51.1	55.2	57.2	59.5	56.2	59.8	55.4	59.1	33.7[c]	57.4	54.2	58,4	58,4	59,9	*mean*	**55.1**
**PrEP use**																	
Time in study when eGFR <60 mL/min/1.73 m^2 (months)	48	0[b]	30	46	11	38	59	1	43	1	16	50	1	36	26		
PrEP use when eGFR <60 mL/min/1.73 m^2 (months)	Daily	Daily	ED	Daily	Daily	Daily	Daily	Daily	''	Daily	Daily	ED	ED	ED	Daily	*N daily(%)*	**9 (75.0%)**
**Variables that may influence kidney function**																	
Medication use[a]	No	No	No	No	Yes, NN	Yes, NN	Yes, PN	Yes, NN	''	Yes, NN	Yes, NN	Yes, NN	No	No	Yes, NN	*N yes(%)*	**7 (58.3%)**
Diabetes mellitus[d]	No	No	No	No	No	No	No	No	''	No	No	Yes	No	No	Yes	*N yes(%)*	**1 (8.3%)**
Cardiovascular disease[d]	No	No	No	No	No	No	Yes	No	''	Yes	No	Yes	No	No	Yes	*N yes(%)*	**4 (33.3%)**

AMPrEP: Amsterdam PrEP Demonstration Project; eGFR = estimated glomerular filtration rate; ED = event-driven; P = participant; 2nd = second measure of a participant below eGFR 60 mL/min/1.73 m^2;
[a] PN = (potentially) nephrotoxic), NN = not-nephrotoxic; [b]enrolment visit, after one month eGFR was >60; [c]measurement after 12 months was >60; [d]deduced from reported medication use by participant.

Supplementary Table S5: Descriptives of participants with persistent proteinuria, defined as two or more consecutive study visits with ≥1+ proteinuria (n=8) among AMPrEP study participants, Amsterdam, the Netherlands, 2015-2020

	P1	P2	P3	P4	P5	P6	P7	P7;2nd	P8	P8;2nd	P8;3rd	P8;4th		Total
**Demographic characteristics**														
Age at baseline (years)	24	37	43	48	53	64	66	66	67	67	67	67	*mean*	**50.3**
Self-reported ethnicity	white	white	white	mixed	white	white	white	white	white	white	white	white		
**Proteinuria measurements**														
Amount of proteinuria when persistent (urine dipstick)	1+	1+	1+	1+	1+	2+	1 to 2+	2+	1+	1+	1+	1 to 2+		
Time in study when persistent proteinuria (months)	34.4	35.5	26.4	34.6	36.9	5.8	2.7	16.8	5.8	14.5	26.3	44.2	*mean*	**25.7**
Duration of proteinuria (months)	3 to 6	3 to 6	3 to 6	3 to 6	3 to 6	3 to 6	9 to 12	3 to 6	3 to 6	3 to 6	6 to 9	6 to 9		
Amount of proteinuria at first measure (urine dipstick)	1+	Neg	Neg	Trace	Neg	Trace	1+	1+	Trace	Trace	Trace	Trace	*N 1+ (%)*	**2(25%)**
**PrEP regimen** (at moment of persistent proteinuria)	Daily	Daily	Daily	Daily	Daily	Daily	ED	ED	Daily	Daily	Daily	Daily	*N daily (%)*	**7(87.5%)**
**Variables that may influence kidney function**														
eGFR (measured with CKD-EPI) around proteinuria	124	110	84	106	79	89	84	95	96	95	91	95	*median*	**95**
Medication use[a]	No	No	Yes, NN	Yes, PN	Yes, NN	Yes, NN	Yes, NN	Yes, NN	Yes, NN	Yes, NN	Yes, NN	Yes, NN	*N yes (%)*	**10(83.3%)**
Diabetes mellitus[d]	No	No	No	No	No	No	No	No	No	No	No	No	*N yes (%)*	**0**
Cardiovascular disease[d]	No	No	No	No	No	Yes	Yes	Yes	Yes	Yes	Yes	Yes	*N yes (%)*	**3(37.5%)**

AMPrEP: Amsterdam PrEP Demonstration Project; eGFR = estimated glomerular filtration rate; ED = event-driven; P = participant; 2nd = second episode of persistent proteinuria; [a] PN = (potentially) nephrotoxic; NN = not-nephrotoxic; [d]deduced from reported medication use by participant.

STROBE Statement—checklist of items that should be included in reports of observational studies

	Item No	Recommendation	Page
**Title and abstract**	1	(*a*) Indicate the study's design with a commonly used term in the title or the abstract<br><br>*Renal outcomes over the course of five years of oral HIV pre-exposure prophylaxis using tenofovir disoproxil/emtricitabine among men who have sex with men*	1
		(*b*) Provide in the abstract an informative and balanced summary of what was done and what was found<br><br>*Methods*<br>*We included men who have sex with men (MSM) from the Amsterdam PrEP demonstration Project (AMPrEP; 2015-2020) at the Public Health Service of Amsterdam who had had at least two creatinine measurements. Participants could choose between daily or event-driven PrEP use. Plasma creatinine was measured at PrEP-commencement and annually thereafter. Kidney function was calculated as the estimated glomerular filtration rate (eGFR) using the CKD-EPI2021 formula.*<br><br>*Results Among 351 participants analyzed (median age 41 years, interquartile range [IQR]=33-49), mean eGFR at PrEP-commencement was 100mL/min/1.73m2 (SD=14). During a median follow-up of 54.2 months (IQR=47.0-57.6), eGFR decreased 0.30 per year (95%CI=-0.59,-0.01). We observed lower mean eGFR over time in those using daily compared to event-driven PrEP (-3.05, 95%CI=-3.95,-2.15), and in older participants (-5.75 per 10 years, 95%CI=-6.70,-4.80). Daily PrEP users had an average decline in eGFR of 0.57 mL/min/1.73 m2/year (95%CI=-1.06 to -0.08), while there was no statistically significant decline in event-driven PrEP users (p for interaction=0.30). Twelve participants (3.4%) had an incident eGFR <60 during follow-up, none of which persisted.*	2
**Introduction**			
Background/rationale	2	Explain the scientific background and rationale for the investigation being reported:<br><br>*There are several crucial barriers that prevent not only people from using PrEP, but also practitioners from prescribing PrEP. One such example is the high frequency of regular medical follow-ups (3), which includes screening for kidney damage (4). TDF may cause renal tubular toxicity, in particular of the proximal tubule (5). Several case reports and cohort studies have shown an association between cumulative TDF exposure in HIV treatment and nephrotoxicity (5). Although TDF/FTC use for PrEP is considered safe and well-tolerated, some studies have also shown a statistically significant decline in estimated glomerular filtration rate (eGFR), yet was reversible after discontinuation of PrEP (6). A recently published meta-analysis found that among 14,368 PrEP users, 2.4% had a decline in eGFR to <60 mL/min/1.73m2 after PrEP-commencement (7). The risk of a clinically*	3

		significant decline in kidney function is known to increase with age, with the highest risk in people above the age of 50. PrEP guidance from the World health Organization (WHO), updated in July 2022, have therefore suggested that kidney function measurement can be considered optional in individuals aged 30 years and younger (8). Despite this recommendation, many of the previous studies have limited follow-up (i.e., median of only 10 months) and no data were available on daily versus event-driven PrEP use. Long-term data are needed to comprehensively address the role of cumulative PrEP exposure in the development of adverse kidney events.	
Objectives	3	State specific objectives, including any prespecified hypotheses<br><br>*The aim of this study was then to assess the association between kidney function and PrEP use over five years of oral PrEP use among daily and event-driven PrEP users in the Amsterdam PrEP demonstration project (AMPrEP) prospective cohort.*	3
**Methods**			
Study design	4	Present key elements of study design early in the paper<br><br>*AMPrEP was a prospective, longitudinal, open-label study with recruitment between August 2015 and May 2016, and follow-up until December 2020.*	4
Setting	5	Describe the setting, locations, and relevant dates, including periods of recruitment, exposure, follow-up, and data collection<br><br>*AMPrEP was a prospective, longitudinal, open-label study with recruitment between August 2015 and May 2016, and follow-up until December 2020.*	4
Participants	6	(*a*) *Cohort study*—Give the eligibility criteria, and the sources and methods of selection of participants. Describe methods of follow-up<br><br>*(…) participants were eligible for inclusion if they were HIV-negative MSM or transgender persons, aged ≥18 years, and reported at least one risk factor for HIV acquisition in the six months prior to the PrEP screening visit. Exclusion criteria were repeatedly having an eGFR <60 mL/min/1.73 m2 (according to the Cockcroft-Gault formula), or concurrent use of nephrotoxic medication. Participants attended follow-up visits every three months. Restarting participation was allowed. (…)*<br><br>*For the present study, all participants of the AMPrEP study were included. Participants were excluded from analyses if they discontinued or were lost to follow-up before creatinine measurement at the 12-month visit, and did not restart.*	4
Variables	7	Clearly define all outcomes, exposures, predictors, potential confounders, and effect modifiers. Give diagnostic criteria, if applicable<br><br>*We used baseline demographic data (i.e., age, gender identity, self-declared ethnicity and socio-economic status). PrEP regimen, categorized as daily or event-driven PrEP, was included as a time-varying variable. We also used data of factors that could potentially influence kidney function, all recorded*	4-5

		annually: the Alcohol Use Disorders Identification Test (AUDIT, in which a score of eight or higher indicates a possible alcohol use disorder (10)), recreational drug use (e.g., amphetamine, cannabis, cocaine, GHB, XTC/MDMA) if used during sex, and the Drug Use Disorders Identification Test (DUDIT, in which a score of eight or higher indicates a possible drug use disorder (11)). Co-medication, including those acquired over the counter, were collected at every study visit. We tested for hepatitis C virus infection annually until December 2016, and every six months thereafter. We measured plasma creatinine at baseline and annually, and performed dipstick urinalysis (UroColorTM 3, SD, Korea) to measure proteinuria at every study visit. Persistent proteinuria was defined as two or more consecutive measurements with ≥1+.	
Data sources/ measurement	8*	For each variable of interest, give sources of data and details of methods of assessment (measurement).<br><br>*We categorized concomitant medication use as follows: nephrotoxic, non-nephrotoxic, or no comedication. Medication was considered to be nephrotoxic if the Liverpool HIV drug interactions database indicated a potential effect on kidney function (12). Medication was considered to be non-nephrotoxic if the database indicated a weak effect. If the medication was not registered in the Liverpool HIV database, the Dutch medication database (Farmacotherapeutisch Kompas) was consulted (13). Participants who were treated with antihypertensives, diuretics, beta-blockers, anti-arrythmics, anticoagulants or lipid-modifying agents were considered to have cardiovascular disease. Participants who reported use of insulin or oral blood glucose lowering agents were considered to be diabetic.*<br><br>*Dried bloodspots (DBS) were collected to measure intraerythrocytic tenofovir diphosphate (TFV-DP) concentrations at 12, 24 and 48 month visits. A detailed description of the laboratory methods has been published before (14). Good adherence was defined as a TFV-DP concentration of ≥700 fmol/punch (corresponding to using ≥4 tablets per week on average) (15).*<br><br>*Plasma creatinine concentration (in μmol/L) was determined using an enzymatic method at the C8000 platform. We calculated eGFR using the CKD-EPI 2021 formula (16). Kidney function stages were defined as normal (eGFR ≥ 90 mL/min/1.73 m2), mildly impaired (eGFR 60-89 mL/min/1.73 m2), moderately impaired (eGFR 30-59 mL/min/1.73 m2), severely impaired (eGFR 15-29 mL/min/1.73 m2) and kidney failure (eGFR <15 mL/min/1.73 m2) following the guidelines from the Kidney Disease Improving Global Outcomes Group (KDIGO) (17).*<br><br>*We measured proteinuria as negative, trace (~10 mg/dL), 1+ (~30 mg/dL), 2+ (~100 mg/dL), 3+ (~300 mg/dL) or 4+ (~1000 mg/dL).*	5-6
Bias	9	Describe any efforts to address potential sources of bias<br><br>*We performed several sensitivity analyses. To assess the possible effect of selective loss to follow-up related to kidney function, we reran the analysis of determinants of eGFR among participants with at least five years of follow-up. As participants switching PrEP regimens could have different changes in eGFR, depending on cumulative exposure to TDF/FTC during*	7

		follow-up, leading to potential bias, we also reran the analysis among participants who never switched regimens.	
Study size	10	Explain how the study size was arrived at (if applicable)<br>n/a	
Quantitative variables	11	Explain how quantitative variables were handled in the analyses. If applicable, describe which groupings were chosen and why<br><br>*We also used data of factors that could potentially influence kidney function, all recorded annually: the Alcohol Use Disorders Identification Test (AUDIT, in which a score of eight or higher indicates a possible alcohol use disorder (10)), recreational drug use (e.g., amphetamine, cannabis, cocaine, GHB, XTC/MDMA) if used during sex, and the Drug Use Disorders Identification Test (DUDIT, in which a score of eight or higher indicates a possible drug use disorder (11)).*<br><br>*Dried bloodspots (DBS) were collected to measure intraerythrocytic tenofovir diphosphate (TFV-DP) concentrations at 12, 24 and 48 month visits. A detailed description of the laboratory methods has been published before (14). Good adherence was defined as a TFV-DP concentration of ≥700 fmol/punch (corresponding to using ≥4 tablets per week on average) (15).*<br><br>*Plasma creatinine concentration (in µmol/L) was determined using an enzymatic method at the C8000 platform. We calculated eGFR using the CKD-EPI 2021 formula (16). Kidney function stages were defined as normal (eGFR ≥ 90 mL/min/1.73 m2), mildly impaired (eGFR 60-89 mL/min/1.73 m2), moderately impaired (eGFR 30-59 mL/min/1.73 m2), severely impaired (eGFR 15-29 mL/min/1.73 m2) and kidney failure (eGFR <15 mL/min/1.73 m2) following the guidelines from the Kidney Disease Improving Global Outcomes Group (KDIGO) (17).*	5-6
Statistical methods	12	(*a*) Describe all statistical methods, including those used to control for confounding<br><br>*Baseline was defined as the enrolment study visit. Follow-up began at baseline and continued until last study visit (due to: study discontinuation, loss to follow-up or reaching end of study) or HIV diagnosis, whichever occurred first. We compared the characteristics of participants with normal versus impaired (i.e., eGFR<90mL/min/1.73m2) kidney function at baseline using Student's t-test or Mann Whitney U-test for continuous variables and Pearson's χ2 or Fisher's exact test for categorical variables.*<br><br>*We assessed determinants for eGFR using multivariable linear regression with generalized estimating equations (GEE) to correct for repeated measurements. Candidate determinants included PrEP use and risk factors for chronic kidney disease. Time (in study), age at baseline, and having diabetes or cardiovascular disease were selected a priori. Other determinants were selected stepwise: First, we included variables with p<0.25 in univariable analyses. We then removed variables with a p≥0.05 via backward selection. Finally, excluded variables were reconsidered in the preliminary model using forward selection if p<0.05. Because of collinearity between PrEP regimen and TFV-DP concentration, we created two separate*	6-7

		multivariable models, one including PrEP regimen as a co-variate and the other including TFV-DP concentration as a covariate.<br><br>*We modeled the probability of having proteinuria over time using GEE for logistic regression. We included covariates separately to estimate the odds ratio (OR) and its 95% confidence intervals (CI) comparing the odds of proteinuria over time across levels of variables.*<br><br>*We constructed a multivariable model in which we included a priori having diabetes or cardiovascular disease. We then included variables with a p<0.25 in univariable analysis and removed variables with a p≥0.05 in backward-stepwise fashion.*<br><br>*We defined significance as a p-value <0.05. All analyses were performed using STATA Intercooled 17 (STATA Corporation, College Station, TX, USA).*	
		(*b*) Describe any methods used to examine subgroups and interactions<br><br>*Characteristics of participants whose eGFR dropped below 60 mL/min/1.73m2 during study follow-up and of participants who had persistent proteinuria (defined as two or more consecutive visits at which proteinuria was found) were described.*	7
		(*c*) Explain how missing data were addressed<br><br>Missing data were not imputed:<br>*Multivariable analyses were based on complete case analysis.*	7
		(*d*) *Cohort study*—If applicable, explain how loss to follow-up was addressed<br><br>*Follow-up began at baseline and continued until last study visit (due to: study discontinuation, loss to follow-up or reaching end of study) or HIV diagnosis, whichever occurred first.*	6
		(*e*) Describe any sensitivity analyses<br><br>*We performed several sensitivity analyses. To assess the possible effect of selective loss to follow-up related to kidney function, we reran the analysis of determinants of eGFR among participants with at least five years of follow-up. As participants switching PrEP regimens could have different changes in eGFR, depending on cumulative exposure to TDF/FTC during follow-up, leading to potential bias, we also reran the analysis among participants who never switched regimens. Finally, much of the previous research on kidney function with PrEP use involved the Cockcroft-Gault formula (7). To enable comparisons with those studies, we performed the analysis on changes in mean eGFR calculated with this formula.*	7
**Results**			
Participants	13*	(a) Report numbers of individuals at each stage of study—eg numbers potentially eligible, examined for eligibility, confirmed eligible, included in the study, completing follow-up, and analyzed	8

		Of 376 participants who initiated PrEP, 351 had a follow-up plasma creatinine measurement and were included in the analyses.	
		(c) Use of a flow diagram<br>n/a	
Descriptive data	14*	(a) Give characteristics of study participants (eg demographic, clinical, social) and information on exposures and potential confounders<br><br>*Two participants identified as transgender women, 349 as male (Table 1). Median age was 41 years (interquartile range[IQR]=33-49). Most participants self-declared as white (84.9%) and had a university or university of applied sciences degree (n=271, 77%). A small proportion of participants had diabetes or cardiovascular disease (n=8 [2%] and n=48 [14%], respectively). People with diabetes or cardiovascular disease had a significantly higher weight than those without (p=0.001). Most participants did not use any comedication at baseline (n=207 [59%]), while 20 (6%) used potentially nephrotoxic comedications. At baseline, 96 (27%) participants chose event-driven PrEP.*<br><br>*At baseline and before PrEP initiation, mean eGFR was 100 mL/min/1.73m2 (standard deviation [SD] 14). 90 participants (25%) had an impaired kidney function, among whom one participant with an eGFR <60 mL/min/1.73m2. This participant was not excluded, because the eGFR according to the Cockroft-Gault formula used at inclusion, was 79 mL/min, and eGFR during follow-up remained >60 mL/min/1.73m2.*	8
		(b) Indicate number of participants with missing data for each variable of interest<br>n/a	
		(c) *Cohort study*—Summarise follow-up time (eg, average and total amount)<br><br>*Median follow-up time was 54.2 months (IQR=47.0-57.6) and 261 (74.4%) participants had a follow-up duration of at least 48 months.*	9
Outcome data	15*	*Cohort study*—Report numbers of outcome events or summary measures over time<br><br>*Mean eGFR at 48 months was 96 mL/min/1.73m2 (SD 15). 201 participants (57.3%) never switched PrEP regimens. During follow-up 2,096 serum creatinine measurements were performed, with a median number of six (IQR= 6-7) measurements per participant. 5,642 urine dipstick measurements were performed: 5,041 (89%) were negative for proteinuria, 520 (9%) showed a trace of protein and 81 (1%) were positive. The median number of urine dipstick measurements per participants was 18 (IQR=16-19). Among daily PrEP users, median TFV-DP concentration at 3 or 6 months was 1,262 fmol/punch (IQR=997–1,622; n=231), at 12 months 1,351 fmol/punch (IQR=1,103–1,697; n=251), at 24 months 1,288 fmol/punch (IQR=1,005–1,617; n=223), and at 48 months 1,693 fmol/punch (IQR = 1,310–2,252; n = 127).*	9

Main results	16	(*a*) Give unadjusted estimates and, if applicable, confounder-adjusted estimates and their precision (eg, 95% confidence interval). Make clear which confounders were adjusted for and why they were included<br><br>*In univariable analysis, lower eGFR was found among those with: higher age at baseline, longer follow-up, using daily PrEP, and having diabetes or cardiovascular disease. Conversely, higher eGFR was found in those with non-white ethnicity and a DUDIT score≥8 (Table 2). In multivariable analysis including PrEP regimen lower eGFR was associated with daily PrEP (-3.02 mL/min/1.73m2, 95%CI=-3.92 to -2.19) and higher age at baseline (-6.16 mL/min/1.73m2 per 10 years older, 95%CI=-7.17 to -5.14; Table 2). In the multivariable model including TFV-DP concentration rather than PrEP regimen, eGFR was associated with TFV-DP concentration (-0.18 mL/min/1.73m2 per 100 fmol/punch increase, 95%CI=-0.31 to -0.05) and higher age at baseline (-5.77 mL/min/1.73m2 per 10 years older, 95%CI=-6.84 to -4.70).*<br><br>*When assessing change in eGFR over time, we noted a significant decline of 0.57 mL/min/1.73 m2/year (95%CI=-1.06 to -0.08, p=0.02) in daily PrEP users, while no significant changes in event-driven PrEP users (+0.02 mL/min/1.73m2/year, 95%CI=-0.83 to +0.79, p=0.96, p for interaction=0.30; Figure 1). In an analysis stratified by age category, no significant change in eGFR was observed in any age category: age <30: +0.44 mL/min/1.73m2/year (95%CI -0.67 to +1.58); age 30-49: 0.14 mL/min/1.73m2/year (95% CI -0.45 to +0.74); age ≥50 years: -0.22 mL/min/1.73m2/year (95% CI -0.89 to +0.44, p=0.51, p for interaction=0.71; Figure 2). (…)*<br><br>*In univariable analysis, proteinuria was more common in those suing daily PrEP, engaging in drug use during sex, and having diabetes or cardiovascular (Table 3). In multivariable analysis, proteinuria was more common in those using daily PrEP (adjusted odds ratio [aOR] 3.19, 95% CI 1.31 to 7.76) and with diabetes or cardiovascular disease (aOR 5.08, 95% CI 1.92 to 13.45; Table 3).*	9-10
Other analyses	17	Report other analyses done—eg analyses of subgroups and interactions, and sensitivity analyses<br><br>*A sensitivity analysis limited to PrEP users who completed at least five years of follow-up included 54/351 (15.4%) participants and yielded comparable results (Suppl. Figure S1). The sensitivity analysis limited to participants who never switched PrEP regimen (based on 201 participants) also yielded comparable results (Suppl. Figure S2). When using the Cockcroft-Gault formula to calculate the eGFR, associations of age and regimen with eGFR were similar, but the decline in eGFR per year was larger than when using the CKD-EPI formula (-1.25 mL/min/1.73m2/year, 95% CI -2.00 to -0.50, p=0.001 in daily PrEP users), and people with diabetes or cardiovascular disease had a significantly higher eGFR (+11.37 mL/min/1.73m2, 95%CI 3.76 to 18.98; Suppl. Table S2). (…)*<br><br>*In sensitivity analysis including TFV-DP concentrations rather than PrEP regimen, TFV-DP concentrations were not associated with the odds of proteinuria (aOR=1.00, 95%CI 0.97 to 1.03 per 100 fmol/punch higher).*	10-11

		Including only participants who never switched PrEP regimens yielded similar results (Suppl. Figure S2).<br><br>*Twelve participants (3.4%) had an eGFR below 60 mL/min/1.73m2, indicating moderate kidney impairment, at least once during follow-up (Suppl. Table S3). Three participants (0.9%) had an eGFR <60 mL/min/1.73m2 measured twice, although never at two consecutive measurements. Eight participants (2.3%) had persistent proteinuria, but never more than at three consecutive measurements (Suppl. Table S4).*	
**Discussion**			
Key results	18	Summarise key results with reference to study objectives<br><br>*In this prospective, longitudinal, open-label demonstration project among 351 PrEP users in Amsterdam, we found that daily PrEP users had a slightly faster decline in eGFR over time and higher odds of developing proteinuria compared to event-driven PrEP users. Older people had lower mean eGFR values of about 10 mL/min/1.73m2 per decade older. In our study population, a decline in eGFR below 60 mL/min/1.73m^2 never persisted and, after adjustments for risk factors related to kidney disease, proteinuria during follow-up was not associated with tenofovir concentrations.*	11
Limitations	19	Discuss limitations of the study, taking into account sources of potential bias or imprecision. Discuss both direction and magnitude of any potential bias<br><br>*Nevertheless, this study has some limitations. First, the study population consisted mostly of white cisgender males with a median age of 41 years, which is not fully representative for the broader population that could benefit from PrEP. Second, information on diabetes and cardiovascular disease was deduced from co-medication. Use of potentially nephrotoxic comedications was low, so power was limited to assess associations. Third, we could not correct for smoking, HbA1c, body mass index and lean body weight, as these measures were not collected. Fourth, for measurement of proteinuria, we used urine dipstick analysis with manual reading, where quantifying is less accurate (17). Cystatin C-based eGFR was also not assessed. In contrast to primary care and hospital settings, for the purpose of screening in low CKD risk populations of a public health setting, dipstick analysis and creatinine-based eGFR are generally acceptable methods (37). Last, following oral PrEP guidelines, we excluded participants with a baseline eGFR <60 mL/min/1.73m2. People with decreased kidney function who are vulnerable to HIV require a tailored approach, using more frequent kidney function monitoring during PrEP follow-up or other PrEP modalities with a better renal safety profile, such as tenofoviralafenamide (38) or cabotegravir (39).*	13
Interpretation	20	Give a cautious overall interpretation of results considering objectives, limitations, multiplicity of analyses, results from similar studies, and other relevant evidence<br><br>*Our finding of a lower eGFR among daily PrEP users compared to event-driven PrEP users is consistent with two observational cohort studies (19-22): ANRS-PREVENIR showed a smaller eGFR decline for event-driven PrEP*	11-13

users compared to daily PrEP users (22) and in iPrEx-OLE the participants had a higher eGFR when taking two pills or less per week compared to seven or more pills per week (19). In addition to those studies we found a significant association between TFV-DP concentration and eGFR, suggesting a dose-response relationship. Because TFV-DP remains longer measurable in in red blood cells (4-6 weeks) than TDF in plasma (several days), and thus is more reflective of long-term adherence; this result is not biased by temporary or short-term adherence prior to study visits.

TDF is primarily associated with proximal tubular dysfunction (23). As a consequence, one would expect eGFR, being a marker of glomerular filtration, not to be affected. Earlier studies suggested, however, that TDF decreases creatinine secretion in the proximal tubule, leading to higher serum creatinine and lower eGFR values (24). The lower eGFR that we observed in daily users may not indicate glomerular impairment, but rather an effect on the proximal renal tubules (25). Proteinuria may also reflect tubular dysfunction (26). We found that daily PrEP was associated with higher odds of proteinuria. However, proteinuria was not independently associated with TFV-DP concentration.

Using the eGFR as a measure for kidney function has limitations: differences in muscle mass also affect creatinine-based formulas like the CKD-EPI, causing an underestimation of the kidney function in muscular people (27). As the Cockcroft-Gault formula estimates creatinine clearance while correcting for body weight, we performed an additional analysis using this formula and we observed similar effects of PrEP on the estimated creatinine clearance. To more accurately analyze treatment effects, the eGFR slope over time can be used (28). We observed a small but statistically significant decline in daily PrEP users, but not in event-driven PrEP users. Thus, there seems to be a small, though real, difference in eGFR between daily and event-driven PrEP users.

We have known for a long time that HIV infection is associated with renal impairment and being on anti-retroviral therapy is associated with a greater eGFR decline and risk of albuminuria (29). Our results indicate negative effects of preventive TDF use on the eGFR and proteinuria are limited compared to effects of TDF use in people with HIV.

Declines to clinically significant levels of eGFR (<60 mL/min/1.73m2) were only observed in 12 participants (3%), and none of those reaching below this threshold remained below 60 mL/min/1.73m2 during follow-up. In previous studies that described clinically significant declines in eGFR after TDF exposure, the study population consisted of people with HIV or chronic hepatitis B virus (30, 31). A possible explanation for this discrepancy could be that TDF is more nephrotoxic in combination with other risk factors, such as HIV and HBV infection, other antiretroviral medications or comorbidities, and a nephrotoxic effect does not emerge in people without these factors. For people with diabetes or cardiovascular disease using PrEP, we did not find a significantly lower eGFR. These comorbidities were, however, associated with proteinuria. Diabetes and cardiovascular disease are known to be associated with proteinuria reflecting glomerular damage and microvascular disease (32, 33).

		The unexpected finding, in the sensitivity analysis using the Cockcroft-Gault formula, that participants with cardiovascular disease or diabetes had a higher eGFR compared to those without such diseases, might be explained by the higher prevalence of excess weight among people with diabetes or cardiovascular disease (Table 1). In the case of excess weight it is advised to calculate the eGFR based on lean body mass instead of weight (34, 35). Because these data were not available, we may have overestimated the eGFR among people with diabetes or cardiovascular disease. Another explanation for the higher eGFR could be glomerular hyperfiltration, which is observed in early stages of diabetes and hypertension (36). This mechanism causes an initial increase in eGFR followed by albuminuria. We observed an association between diabetes and cardiovascular disease and proteinuria, while the eGFR was higher in these groups. The lower value found with the Cockcroft-Gault formula is fitting this phenomenon.<br><br>*The decline in eGFR over time seemed faster in people aged ≥50 years compared to younger people, however not significant. This is in line with a previously reported meta-analysis (7). The observed decline among participants ≥50 years (-0.22 mL/min/1.73m2/year (p=0.51)) was smaller than the reported normal range for ageing (-0.82 to -1.15 mL/min/1.73m2/year (37)), and may not directly pose safety concerns for PrEP use in this age category. Our findings support the modified WHO guidelines stating the eGFR in people aged ≥50 years using PrEP should be monitored annually. For younger PrEP users and individuals without (renal) comorbidities, less frequent or optional monitoring of the kidney function is appropriate. The WHO guidelines advice to make screening optional in individuals <30 years and optional or once around the time of initiation in individuals aged ≤50 without kidney-related comorbidities (8). This lower eGFR screening frequency may prove to be crucial in reducing costs and barriers for prescribing and using PrEP. Depending on available resources is it worth considering systematically screening eGFR at least once at PrEP initiation.*	
Generalisability	21	Discuss the generalisability (external validity) of the study results<br><br>*(…) the study population consisted mostly of white cisgender males with a median age of 41 years, which is not fully representative for the broader population that could benefit from PrEP.*	14
**Other information**			
Funding	22	Give the source of funding and the role of the funders for the present study and, if applicable, for the original study on which the present article is based<br><br>*ZonMw, H-TEAM, RIVM, GGD research funds, Aidsfonds, Amsterdam Diner Foundation, Gilead Sciences, Gilead Sciences Europe Ltd, Janssen Pharmaceuticals, MAC AIDS Fund, ViiV Healthcare.*	15
